# A research and development investment strategy to achieve the Paris climate agreement

**DOI:** 10.1038/s41467-023-38620-4

**Published:** 2023-06-16

**Authors:** Lara Aleluia Reis, Zoi Vrontisi, Elena Verdolini, Kostas Fragkiadakis, Massimo Tavoni

**Affiliations:** 1 RFF-CMCC European Institute of Economics and the Environment, Euro-Mediterranean Center on Climate Change, Milan, Italy; 2 E3Modelling S.A., Athens, Greece; 3 University of Brescia, Department of Law, Brescia, Italy; 4 Politecnico di Milano, Department of Management, Economics and Industrial Engineering, Milan, Italy

**Keywords:** Climate-change policy, Environmental economics

## Abstract

Climate stabilization requires the deployment of several low-carbon options, some of which are still not available at large scale or are too costly. Governments will have to make important decisions on how to incentivize Research and Development (R&D). Yet, current assessments of climate neutrality typically do not include research-driven innovation. Here, we link two integrated assessment models to study R&D investment pathways consistent with climate stabilization and suggest a consistent financing scheme. We focus on five low-carbon technologies and on energy efficiency measures. We find that timely R&D investment in these technologies lowers mitigation costs and induces positive employment effects. Achieving 2 °C (1.5 °C) requires a global 18% (64%) increase in cumulative low-carbon R&D investment relative to the reference scenario by mid-century. We show that carbon revenues are sufficient to both finance the additional R&D investment requirements and generate economic benefits by reducing distortionary taxation, such as payroll taxes, thus enhancing job creation.

## Abstract (web summary)

Timely R&D investment in green technologies lowers mitigation costs with positive employment effects. Carbon revenues are sufficient to finance the additional R&D investment and generate economic benefits.

## Introduction

Achieving the Paris Agreement goal of limiting global warming to well below 2 °C implies peaking anthropogenic emissions as soon as possible through the phase-out of traditional fossil-based energy and the fast deployment of low-carbon and negative-emission technologies. These include renewable energy and carbon capture and storage (CCS)^1,2^. To achieve such a rapid transition, the pace of energy innovation and technology diffusion will have to be scaled up significantly^3,4^. Importantly, a large share of the greenhouse gas (GHG) emissions reductions consistent with 2 °C and below 2 °C scenarios rely on technologies that are not fully market-ready today^5^.

Public research, development and demonstration (R&D) investments play a critical role in fostering technological progress in all sectors, including energy^5,6^. Public investments provide “patient capital”, and allow to overcome the uncertainty which is intrinsic in the process of non-incremental energy innovation^7–9^. The benefits associated with learning-by-research dynamics are particularly important for less mature technologies^10^. Indeed, state-funded investments for innovation, supported by coherent policy frameworks which include supply-side as well as demand-side policies, contributed to the development of cost-competitive low-carbon technologies, such as wind, solar LEDs and batteries for electric vehicles^7,11–13^. The literature supporting this thesis is rich, and results are largely consistent: R&D investments contribute to lowering energy technology costs^14^.

Choices in public R&D funding among several low-carbon technologies at different stages of development—such as Carbon Capture and Storage (CCS), batteries for electric vehicles and advanced biofuels—are instrumental to achieving the transition^15^. Informing such choices requires an understanding of the complex interactions between several low-carbon options, as tailored technology portfolios need to be identified depending on the geographical specificities and natural resource endowments of a given region and country^16,17^. Given the long time horizon which characterizes the energy system and climate-related impacts, decisions on R&D investments need to be optimized intertemporally^18^. The timing of such investment is crucial to increase the likelihood of limiting global warming over the next decades^19^. Furthermore, a non-trivial question relates to the mechanism through which RD&D investments can be financed^20–22^.This latter aspect is overlooked in most integrated modeling assessments of climate stabilization.

This paper provides insights to inform the choice of energy technology portfolios, the timing of R&D investments, and a possible financing mechanism in the context of a rapid energy transition to limit global warming to well below 2 °C. We soft-link two well-established Integrated Assessment Models (IAMs): WITCH^23^—to identify innovation investments for five key decarbonization technologies and on a package of measures promoting energy efficiency—and GEM-E3^24^ to study a mechanism for low-carbon R&D investments through the recycling of carbon revenues. The use of a dynamic model (WITCH) allows calculating the optimal level of R&D investment taking into account the time lag with which lower technology costs accrue as a result of research investments^25^. Optimal R&D investments are those which are cost-effective for each region, i.e., the least-cost option maximizing each region’s welfare, with or without a carbon budget constraint or an R&D budget constraint. Through WITCH, we account for the substitution and complementarity of different low-carbon technology options and allow for a full-century definition of the R&D investment pathways. The use of a computable general equilibrium (CGE) model with a detailed representation of the economy (GEM-E3) allows us to study the R&D financing policy and understand the economy-wide competitiveness and employment implications of different investment choices. Specifically, GEM-E3 includes 67 production sectors—10 of which relate to the manufacturing of low-carbon technologies—and a representation of employment dynamics in the labor market.

Our results provide insights on four fronts: global innovation strategies compliant with Paris targets, a feasible option for the financing of R&D through carbon revenues, macroeconomic repercussions of the R&D strategies, and the implications for the global mitigation cost of climate policies.

## Results

Our analysis generates detailed results for different technologies and regions and for each of the scenarios explored. All regional results can be explored and compared using an open access on-line tool we developed (https://datashowb.shinyapps.io/web_shiny/). Here we summarize the main insights emerging from the analysis in terms of (a) global pathways, (b) the financing of additional R&D investments in stringent decarbonization scenarios and (c) regional macroeconomic effects. We also discuss (d) global climate policy costs with optimal R&D strategies.

### Global pathways

**Fig. 1:**
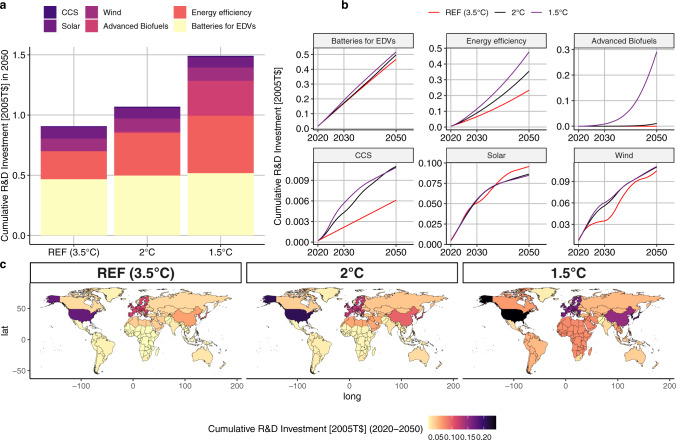
Global Policy pathways provided by the WITCH model. **a** shows the global cumulative R&D investment up to 2050; **b** shows the global cumulative investments per technology from 2020 to 2050; **c** shows the total R&D low-carbon investments for the WITCH regions by scenario.

Figure 1 summarizes our results in terms of global cumulative optimal investment from 2020 to 2050 for each technology (panel a), time profile (panel b) and total cumulative R&D investment in selected regions (panel c). Four main messages emerge.

First, while optimal R&D investments in all scenarios fund a diverse portfolio of technologies, the relative importance of a given technology is different depending on the stringency of the carbon budget. In all three scenarios, the biggest share of investments is allocated to batteries for EDVs and energy efficiency. In the REF scenario and in the 2 °C target, wind and solar represent respectively the third and fourth largest R&D investments. In the 1.5 °C target, advanced biofuels is the third most funded technology, before wind and solar, in line with historical shares^5^. For CCS and Wind, the results are more sensitive to the presence of a carbon budget than to the magnitude of the budget itself. The CCS share of R&D investments remains low compared to other technologies; this result is discussed more in detail below.

Second, for more mature technologies, the overall optimal R&D low-carbon investments in stringent decarbonization scenarios do not necessarily mean more cumulative R&D investment for each technology, but rather earlier R&D funding (Fig. 1b). Indeed, total cumulative investments for solar and wind over the century in the REF and policy scenarios are similar, i.e., optimal investment does not require large scale-up in a policy scenario as compared to REF (see also Supplementary Figs. 10 and 11 of the Supplementary Information). Two considerations help explain this result. On the one hand, these technologies are characterized by high learning-by-researching and learning-by-doing rates. On the other hand, as we discuss below, in stringent decarbonization scenarios these technologies compete with CCS. Importantly, however, R&D investments for mature technologies in both climate policy scenarios are carried out earlier rather than later. In the case of solar, for instance, imposing a more stringent climate policy results in a displacement of solar R&D investments from the later years to 2030. Similar dynamics are apparent, to some extent, for wind. This result, emerging from a perfect foresight model (WITCH), confirms that R&D is a key component of the climate policy portfolio and that, for more mature technologies, it is not only its amount but also the timing of such investment that matters in achieving more stringent targets at lower costs. Note that the two–factor learning curve formulation included in WITCH contributes to achieving this result (see Equation 3 of Supplementary Information section 1). As mentioned above, the two-factor-learning curve formulation accounts for cost decreases arising from both R&D investments (through the knowledge stock) and from increasing deployment (through cumulative capacity). At the beginning of the century, optimal R&D investments are larger and result in lower installation costs, consequently leading to faster deployment. This increase in installed capacity, in turn, further lowers installation costs. The cumulative effect of these dynamics is more pronounced for technologies with high learning-by-doing rates, such as solar and, to a certain extent, wind (see Supplementary Fig. 6 of the Supplementary Information section 3). Importantly, as we discuss below, in stringent decarbonization scenarios these technologies compete with CCS.

Third, for less mature technologies, there are substantial differences in the optimal R&D investments in the REF as opposed to the decarbonization scenarios. CCS is the least mature of the technologies analyzed here, and one that has not yet registered large R&D investments relative to the other technologies. The fact that returns on R&D investment are calibrated with historical data and that for less mature technologies a successful technological outcome is more uncertain explains the lower investment levels compared to those of other technologies. Yet, investments in CCS in 2030 are double and 2.6 times larger in the 2 °C and 1.5 °C scenarios, respectively, as compared to REF. This in turn gives rise to a rapid decrease in the cost of fossil energy with CCS, illustrating the importance of this technology option to support the rapid emission reductions in the early years. Indeed, the availability of low-cost fossil energy with CCS has important implications for the time profile of energy demand from other low-carbon technologies. Specifically, it translates into slightly lower deployment of renewables, even if the R&D investments have reduced the cost of solar and wind. While scenario literature suggests that CCS is an important component of reaching the 2 °C and 1.5 °C decarbonization targets^26^, our study highlights that CCS technologies compete with renewable energy sources. Differently from CCS, batteries for vehicles see only a timid increase in R&D investment in the presence of a climate policy, as compared to REF. However, given that this technology has the highest learning-by-researching and learning-by-doing rates even small increases in investments and capacity lead to great cost reductions (see Section 4 of the Supplementary Information for sensitivity to learning rates).

Fourth, total cumulative R&D investments are heterogeneous across different regions in the world (Fig. 1c). In all scenarios, the highest contributors to R&D investments are the USA, the EU and Japan and South Korea (jpnkor). When climate policy is implemented (2 °C and 1.5 °C) all regions increase their R&D investments (Supplementary Fig. 19 of the Supplementary Information). With the exception of the USA, Russian and the former Soviet Union and the MENA region, the change in R&D investments required to achieve 2 °C relative to REF is lower than the one required to achieve 1.5 °C relative to 2 °C. That is, reaching the 1.5 °C target requires a deeper change in energy systems than reaching the 2 °C target (Supplementary Fig. 19 of the Supplementary Information). Reaching the 1.5 °C target entails a 64% increase in global cumulative R&D investments as compared to the 18% needed in 2 °C. In the 1.5 °C scenario the global R&D average (2020–2050) investment estimate is 18.8 2005$ billions. According to UNEP^27^ the estimation of clean R&D spending in the COVID-19 recovery packages is 28.9 2005$ billions, that is 65% of the annual estimated needs to reach 1.5 °C.

Comparing the current public investments provided by^13^ for the regions where data is available, in the USA, Oceania, Japan and South Korea, the EU, and Canada the estimated low-carbon R&D investment needs in 2050 represent more than double of the current low-carbon R&D investments in terms of Share of GDP (Supplementary Fig. 19 of the Supplementary Information section 10). Note that R&D in EDV is excluded from this comparison as^13^ reports data on public expenditures while our analysis of EDVs includes both private and public data, as explained earlier, so such a comparison would be misleading. In China, Japan and South Korea, Southeast Asia, Latin America and sub-saharan Africa reaching 1.5 °C implies at least doubling the R&D investment with respect to REF. These increases are related to investments in advanced biofuels (Supplementary Fig. 14 of the Supplementary Information). The EU and the USA have high R&D investments in batteries for EDVs in the REF scenario as well as in low-carbon scenarios. Conversely, in China, the MENA region, and to some extent the reforming economies, R&D investments in this technology are triggered only by stringent climate policy (Supplementary Fig. 10 of the Supplementary Information). CCS R&D investments see important increases in Latin America (except Brazil), Canada, USA, China and the reforming economies (Supplementary Fig. 12 of the Supplementary Information).

### Financing low-carbon R&D through carbon revenues

Financing the energy transition is a challenge^21^ state that R&D is only a small share of the overall financing requirements of the low carbon transition. Our estimated additional low-carbon R&D investment requirements to reach 2 °C (1.5 °C) for low- and mid-income countries represent 3.0–3.2% (12–13%) of the climate finance estimated by the OECD in 2021^28^ (see Supplementary Fig. 22 of the Supplementary Information).

**Fig. 2:**
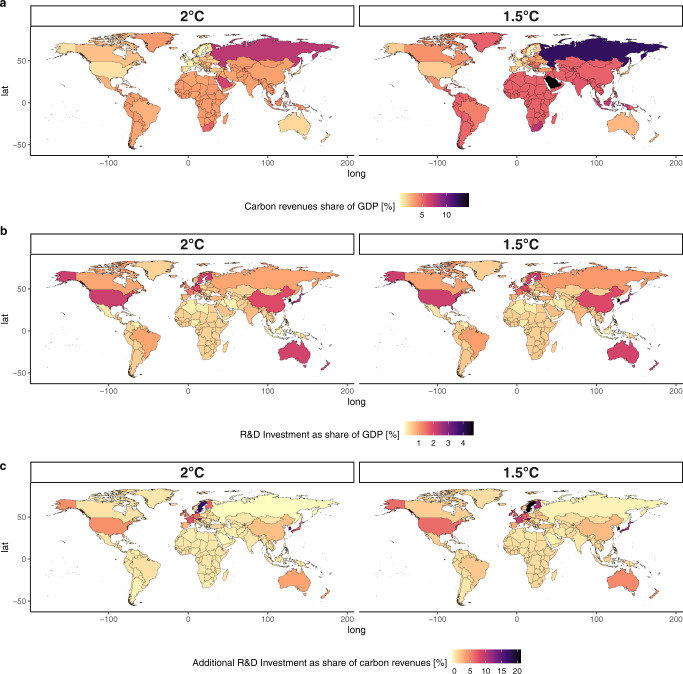
R&D financing. **a** shows carbon revenues as a percentage share of GDP by country for the 2 temperature targets; **b** shows the all sector R&D Investment as a share of GDP; **c** shows the additional all sector R&D Investment expressed as a percentage share of carbon revenues; source: GEM-E3 model. All the panels refer to the year 2050.

Here, we do not consider the option of international financial transfers, but rather focus on domestic financing through dedicated climate fiscal tools. Carbon revenues constitute a fiscal tool that can be redirected to the economy to facilitate double dividends, provide enabling conditions for the low-carbon transition, enhance innovation or remove existing economic distortions. In our assessment with the GEM-E3 model, we assume that carbon revenues will be used to finance the additional R&D needs for each policy scenario as compared to the REF scenario, and that any remaining carbon revenue is directed to lowering payroll taxes. Figure 2 summarizes the results with respect to the financing of the R&D investments.

Panel a of Fig. 2 shows that carbon revenues as a percentage of GDP vary significantly by country in both carbon budget scenarios, depending on the regional carbon intensity and the respective effort required to achieve the emission reductions. Overall revenues reach 2.5% and 4.1% of global GDP in 2050 in the 2 °C and 1.5 °C scenarios, respectively. In carbon-intensive fossil fuel producing countries, such as Saudi Arabia and Russia, carbon revenues are a much higher share of GDP, reaching 14% in 2050. Figure 2b shows that estimated total R&D investments in all sectors and technologies, including those focus of this study, as a share of GDP in 2050 are similar in the two temperature target scenarios. Importantly, in all regions the estimated total R&D needs, including low-carbon, represent a share of GDP in line with current levels, with the exception of Argentina and South Korea (Supplementary Fig. 20 of the Supplementary Information). In oil exporting countries, Russia, and sub-saharan Africa R&D investment represents a lower GDP share than the current (2019) share of fossil fuels subsidies (Supplementary Fig. 20 of Supplementary Information). In this case, a redirection of government funds would suffice to finance the R&D needed. Achieving stringent policy scenarios requires limited increased R&D investments, as explained above. Figure 2c shows that for the majority of regions, this additional R&D funding is only a small share of carbon revenues, ranging from 0.5% in Argentina to 21% in Sweden in the 1.5 °C scenario. The remaining carbon revenues are redirected to the economy via a reduction of payroll taxes. Our analysis shows that, at the global level, additional R&D investments between the REF and the low-carbon scenarios can be financed using 2% of global carbon revenues. Overall, our results confirm that R&D investments are an efficient strategy for carbon revenue recycling. Once this is combined with the reduction of labor-related taxation, wider co-benefits can emerge. Using carbon revenues to finance low-carbon R&D constitutes an effective way to implement the “polluter pays” principle in practice, allowing even the poorest regions to ensure some financing transactions from the polluters to the clean technology sectors. The optimal trajectory of R&D investments is well aligned with the availability of carbon revenues, given that high carbon revenues are expected in the early years of the mitigation action, when R&D should be more intense. Once the economy decarbonizes, carbon revenues become low, but R&D requirements are less pronounced.

Importantly, our scenario implementation considers only the financing of additional R&D investments through carbon revenues and not the entire amount which also includes the R&D investments of the REF scenario. Conversely, the rest of the R&D investments are financed through the government budget, as in the REF scenario. Financing the entire R&D investments (and not only the additional scenario-related funds) through carbon revenues would amount to 57 and 35% of global carbon revenues in 2050 in the 2 °C and 1.5 °C scenarios, respectively. However, in several countries, carbon revenues would not be sufficient to finance the entire R&D investments, particularly in the 2 °C scenario.

We note that the GEM-E3 analysis does not include the additional R&D investments for energy efficiency improvements that are described in the previous section due to methodological limitations in linking the two models in this respect. To address this limitation, we carried out post-processed calculations to assess if energy efficiency investments could also be financed by the carbon revenues. Indeed, as shown in Supplementary Fig. 16 of the Supplementary Information, all additional R&D investments (i.e., including energy efficiency) can be financed by the carbon revenues. On a regional level, financing the entire R&D investments requires a maximum of 18% (22% in the 1.5 °C scenario) of carbon revenues in 2050. Accounting for energy efficiency R&D investments still allows a large share of carbon revenues to reduce payroll taxes.

### Regional macroeconomic effects in major economies

**Fig. 3:**
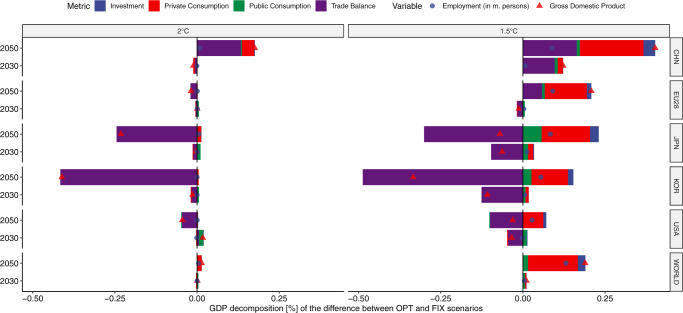
Macro-economic indicators resulting from the R&D financing strategy. Indicators are shown for selected regions and for both climate targets, source GEM-E3 model.

R&D investments lower the costs of clean technologies; this in turn reduces the cost of mitigation. These cost reductions improve overall global economic activity as well as the competitiveness of certain regions. Figure 3 shows regional macroeconomic implications of the optimal R&D strategies in relation to the non-optimal (FIX) ones for both temperature target scenarios estimated with the GEM-E3 model. In the 2 °C scenario, GDP impacts are small as the increase of R&D investments from REF/FIX levels by 2050 is limited for all technologies considered by GEM-E3 model, with the exception of CCS (Fig. 1 panel b). Note that energy efficiency R&D investments are not considered in the GEM-E3 model runs, as discussed above. Globally, small GDP gains are registered in 2030 and 2050. Conversely, at the regional level economic results are more diverse and driven mainly by changes in competitiveness for clean technology goods. For example, Japan shows a GDP loss of 0.23% and South Korea of 0.4% both from the respective 2 °C_FIX levels, due to lower exports of batteries to the benefit of China, while the latter registers a 0.18% increase in GDP in 2050 due to the higher export levels (Supplementary Fig. 10 in the Supplementary Information).

GDP gains are more pronounced in the more stringent 1.5 °C scenario. Higher R&D investments increase global GDP by 0.2% relative to the REF scenario in 2050 as the low-carbon transition becomes less costly. China is among the countries which benefit most, with an increase of 0.4% in GDP in 2050 relative to 1.5 °C_FIX levels. This arises from the fact that the production of batteries and electric vehicles becomes more competitive and thus increases the supply towards both the domestic and international markets. Similarly, the EU28 sees a 0.25% increase in GDP in 2050, due to both higher export levels (particularly of advanced biofuels) and to higher private consumption levels. In particular, the lower price of electricity and transportation resulting from lower technology costs allows for the consumption of more goods and services as compared to 1.5 °C_FIX levels. Changes in the global advanced biofuels markets account for most of the positive macroeconomic impacts of countries such as Argentina, Indonesia and Brazil, which show GDP gains of 1.2%, 0.7% and 0.13% compared to 1.5 °C_FIX levels in 2050, respectively.

Globally, employment effects are positive as economic activities increase. The induced effects on employment are driving results despite the lower availability of climate-related fiscal revenues to reduce payroll taxes due to the financing of R&D. On a regional level, employment levels are higher than the corresponding FIX levels, even in cases where economic activity falls. Our estimates suggest that financing R&D activities brings a positive direct multiplier effect due to the high labor intensity of the R&D process.

### Global climate policy costs with optimal R&D strategies

**Fig. 4:**
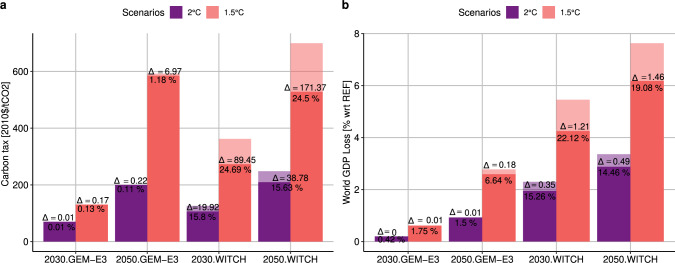
Global cost metrics of the different policy scenarios for both models. **a** refers to GEM-E3 and **b** refers to WITCH. Shaded bars represent the FIX scenarios with frozen R&D investments while full colored bars represent the optimal scenarios. The delta (Δ) represents the value difference between the two. A positive value means higher GDP loss or carbon price in the FIX scenario with frozen R&D investments.

Optimal R&D strategies improve the feasibility of climate policies by lowering technology costs and thus reducing the level of the required carbon tax and the associated global mitigation cost (i.e., GDP loss as shown in Fig. 4). This result is robust across both models and is more pronounced in the most stringent climate target of 1.5 °C, where emission cuts are faster and deeper: in these cases policy costs drop by 7–19% by mid-century. GEM-E3 results indicate that each $ of R&D invested corresponds to a 1.64 and 8.01 2005$ increase in GDP for a 2 °C and 1.5 °C policies in 2050, respectively. Similarly, for each 2005M$ of R&D investment, 7 and 96 additional people are employed in 2050 for a 2 °C and 1.5 °C policies, respectively.

## Discussion

In this paper we show how optimization decision tools can be used to support policy makers in choosing optimal R&D strategies and in assessing the macroeconomic implications of R&D investments. We implement a consistent multi-model framework to study optimal R&D strategies to reach the Paris Agreement’s long-term climate stabilization targets. This innovative approach combines the strengths of two well-established integrated assessment models. The WITCH model provides intertemporal optimization of the R&D investments; the GEM-E3 model enables a detailed macroeconomic assessment of R&D pathways and respective financing mechanisms.

Our results can be summarized as follows. Independent of the stringency of the decarbonization target, the biggest share of investment in R&D goes to batteries for cars and energy efficiency up to 2050. Investments in R&D should start early–scaling-up in 2025–and ramp up gradually in order to allow a less costly transition. This is consistent with previous studies^29^, where an initial investment peak is needed to start the transition and then R&D investment can slowly decrease as the learning-by-doing effect takes over in the two-factor learning curve equation. Energy efficiency R&D investments grow steadily throughout the century, as these are applied gradually to all the energy demand sectors contributing to reducing total energy demand. The relative importance of other low carbon technologies differs depending on the stringency of the carbon budget: solar and wind investments are higher in the less stringent decarbonization target, while CCS is higher in the more stringent climate scenario.

For technologies that are already at the deployment stage, such as wind and solar, the timing of investments is pivotal. We find that it is the early investment action rather than high investment levels that matter most to reach the Paris Agreement targets. Conversely, for technologies that are currently less market-ready, such as CCS, achieving stringent decarbonization targets implies an increase in R&D levels. The relative importance of different technologies in different regions changes depending on the stringency of the decarbonization target, and the dynamics of competition between technologies emerge. On the one hand, the wider the portfolio of technologies supported through R&D, the lower the global costs of compliance with the long-term targets of the Paris Agreement. On the other hand, investing in less mature (substitute) technologies such as CCS reduces the need to invest in more mature renewable options.

Overall, optimal R&D strategies increase the feasibility of the climate targets, lowering carbon prices and the costs of mitigation. These results are robust across both models and show the extent to which R&D is an enabler of climate policies. GDP gains are more pronounced in the more stringent 1.5 °C scenario. On a global level, each 2005$ of R&D invested corresponds to a 1.64 and 8.01 2005$ increase in GDP for 2 °C and 1.5 °C policies in 2050, respectively. Similarly, for each 2005M$ of R&D investment 7 and 96 additional people are employed in 2050 for 2 °C and 1.5 °C policies, respectively. The 2 °C employment effectiveness estimates are in line with the IMF (2021)^30^ estimates of the recovery packages. With the exception of South Korea, our estimated total R&D investment needs in 2050 are lower than the current total R&D investments in terms of share of GDP. However, low-carbon innovation must increase substantially in all OECD countries to reach 1.5 °C (Supplementary Fig. 20 of the Supplementary Information). To put the level of investment requirements into context, we show that, for oil exporting countries (e.g., Russia), the R&D investments needed are lower than current fossil fuel subsidies.

Finally, optimal R&D efforts to meet the 2 °C and 1.5 °C targets can be financed via carbon revenues at lower global costs and with positive effects on employment. Carbon revenues enable an application of the ‘polluter pays’ principle while ensuring double dividends occurring from the removal of pre-existing distorting taxation, such as payroll taxes. This approach mitigates the problem of uncertainty related to less mature technologies, given that public R&D, like any other public funding, obtained from the carbon revenues, represents “patient” capital, which is often required/used to cover more risky and longer term investments, including those of less mature low-carbon technologies such as CCS. Especially if they create double dividends as in the case of low-carbon technologies. Redirecting the carbon revenues for the financing of R&D investments allows even the poorest regions to ensure the financing of innovation strategies, while avoiding transfers to carbon-intensive investments. The ideal trajectory of R&D investments is well aligned with the availability of carbon revenues, given that high carbon revenues are expected in the early years of the mitigation action when R&D should be more intense. Once the economy decarbonizes, carbon revenues become low, but R&D requirements lower as well. Since we only assess one financing method, further research on the low-carbon R&D financing strategies is needed, including strategies to foster low-carbon R&D investments in countries with low financing capacity. Nonetheless, the study points to the centrality of innovation strategies in ensuring the decarbonization is fast and equitable.

## Methods

### RD&D investments and technologies

Our analysis focuses on five decarbonization technologies—batteries for vehicles, advanced biofuels, solar, wind power, CCS—and on a package of measures promoting energy efficiency. This choice is dictated by two reasons. First, these technologies have important mitigation potential in key sectors of the economy, including the power sector, transport and energy demand services ^31^. Second, we focus on these specific technologies because they are modeled as part of the IAMs we rely on and because detailed and long datasets on R&D investments and patents are available for model calibration. This naturally implies that some other potentially very relevant technologies are not considered as part of our study. These include, for example, Direct Air Capture and hydrogen. The former is not included because its innovation dynamics are currently too uncertain and not easily modeled ^32^. The latter, while being less uncertain, raises significant concerns given its high energy-intensive production process, which may indeed invalidate its potential relevance for a sustainable low-carbon transition ^33^. We, therefore, do not consider the (currently highly speculative) possibility that the large-scale availability of DAC technologies or hydrogen may have the potential to shift investments away from renewables and other low-carbon technologies ^34^. In any case, note that DAC relies on CCS technologies, which are included in the R&D investment portfolio considered in our analysis.

The estimates we generate relate to public R&D, with the exception of batteries for Electric Driven Vehicles (EDV’s) where most of the R&D investments have been carried out by the private sector.

### Models

A general description of the models used in this analysis can be found ^23^(WITCH) and ^24^ (GEM-E3). A detailed description of how both models depict technological change dynamics is provided in Supplementary Information sections 1 and 2. Here we mention two main features of the models which are relevant for this analysis. First, both model formulations include two-factor learning curves, i.e., costs modeled as a decreasing function of installed capacity and investments in R&D ^35^. This allows accounting for two crucial determinants of innovation and associated costs reduction, namely investment in research and of growing markets (i.e., capacity). The former is more relevant in the early stages of innovation, the latter becomes increasingly important as technologies mature and enter the market ^36^. Particularly, learning-by-doing can lead to technology improvements over time^37^. Indeed, the two-factor learning curve captures cost decreases arising from both the knowledge stock, through R&D investment, and from technology diffusion through higher deployment. Second, both models also integrate knowledge spillovers to reflect the role of foreign knowledge in domestic innovation^23,38,39^. This key feature allows to model R&D investment decisions that build on the positive knowledge externality arising from R&D ^40^.

All the learning rates used were taken from literature, as shown in Table 1. The learning rate values are somewhat normative. In addition, at this level of technological detail, learning-by-doing and learning-by-researching rates are often estimated separately, i.e., by different studies focusing on either the former or the latter. This raises potential concerns of at least partially double counting. Given all this, it is therefore crucial to perform sensitivity analysis on these learning rate parameters to validate the robustness of results and to highlight the extent of potential change to varying assumptions. Sensitivity is presented in Section 4 of the Supplementary Information. This sensitivity shows that, with the exception of advanced biofuels and wind, R&D investments are not very sensitive to changes in learning rates. Advanced biofuels and wind, on the other hand, show sensitivity to changes in their learning rates. For advanced biofuels this only happens in the 1.5 °C scenario, meaning that the stringency of the target is the crucial parameter and learning rates only provoke changes in R&D investment amounts and not so much in deployment. For wind, R&D investments may vary substantially but this remains a low share of the R&D low-carbon investments. The time lag from investment to knowledge generation is five years; the assumed knowledge stocks depreciation rate is 0.05. Both are in line with the available literature. Additionally, we have harmonized the technology floor costs and the initial knowledge stocks.

**Table 1:** List of harmonized learning rates between models

Sector	Learning-by-doing rates	Source	Learning-by-research rates	Source
Advanced biofuels	0.08	Handayani et al. (2019)^46^	0.13	Emmerling et al. (2016)^23^
CCS	0.05	European Commission (2018)^47^	0.03	Verdolini et al. (2018)^48^
Wind onshore	0.06	Louwen et al. (2018)^49^	0.17	Rubin et al. (2015)^50^
Wind offshore	0.1	Louwen et al. (2018)^49^	0.17	Rubin et al. (2015)^50^
Solar PV	0.18	Louwen et al. (2018)^49^	0.12	Rubin et al. (2015)^50^
Batteries	0.2	IEA 2019^12^	0.27	Mayer et al. (2012)^51^

**Fig. 5:**
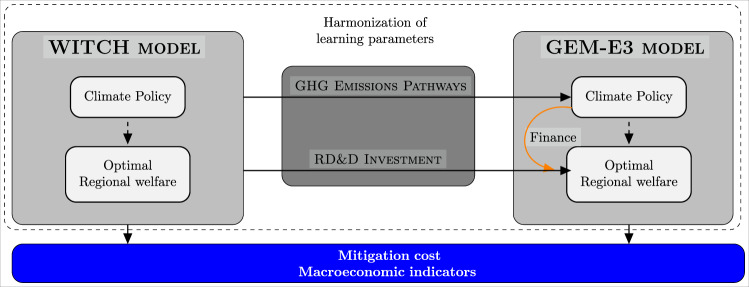
Schematic representation of the modeling framework. The orange arrow represents the revenues from the climate policy being recycled to finance the R&D investments.

Figure 5 summarizes the soft-linking approach adopted in this study, which allows us to combine an estimation of the optimal trajectory for R&D investments with a consistent assessment of financing policies and cost estimations. In particular, the approach enables the calculation of the optimal level of R&D investment taking into consideration all specificities of the innovation process (time lag characterizing the returns to R&D investment, substitution and complementarity of different low-carbon technology options, full-century optimization) while integrating an R&D financing policy via carbon revenues. This allows us to explore and discuss the macroeconomic and employment implications of different investment choices.

The sequencing of our methodology is as follows. First, WITCH calculates the global and regional R&D optimal investment level by technology and climate policy target. This serves as input for the GEM-E3 model. Specifically, to establish a harmonized model framework, WITCH provides the following inputs to the GEM-E3 model: (i) the global CO2 emission pathways, (ii) the R&D knowledge stock by technology and region, (iii) the R&D investment by technology (with the exception of energy efficiency measures) and region. Second, the GEM-E3 model provides a detailed assessment of the funding mechanism to support R&D investments and examines the financial feasibility of the optimal R&D pathways. The GEM-E3 model features high sectoral and regional detail and endogenous bilateral trade in a closed CGE framework. This set up ensures a consistent financing of R&D investment and the assessment of macroeconomic and competitiveness impacts ^41^.

In terms of carbon price levels, the carbon tax is estimated in the WITCH model by iteration until the policy targets are reached, and endogenously in GEM-E3 (to achieve the emission trajectory provided in each scenario).

### Scenarios

The Reference (REF) scenario, which serves as a point of comparison, has the following characteristics: for the WITCH model, it is equivalent to the SSP2 “middle of the road’ baseline scenario as defined in^42^, assuming no increase in policy stringency. The SSP2 scenario has been widely used by IAMs as their baseline reference scenario^42–44^. For the GEM-E3 model, the REF scenario includes current policies as described in ref. ^45^ for the short term, incorporating climate and energy policies legislated as of 2017; population and socioeconomic projections are in line with the European Commission Ageing Report 2019 and the projections by UN and OECD. After 2030, assumptions are consistent with SSP2, as described above.

Table 2 illustrates the policy scenarios modeled in our analysis, whose key characteristics are common to both models. A common climate policy scenario that features a stylized global mitigation action consistent with constraining global average temperature increase by 2100 to well below 2 °C. This is achieved by imposing a carbon budget (CB) of 1460 and 710 GtCO_2_ for the period 2011–2100 (for details see ref. ^2^). Both models simulate two variants of this climate target scenario. The first assumes the enabling of optimal R&D investment (“OPT”); the second does not allow for R&D investment on the selected low-carbon technologies; instead, R&D investments are fixed at the REF levels (“FIX”).

**Table 2:** Scenario definition and description

Scenarios	REF	2 °C	2 °C_FIX	1.5 °C	1.5 °C_FIX
Climate stabilization target	No climate stabilization target (leading to a temperature of 3.5 °C by 2100)	Carbon budget of 1460, consistent with 2 °C average increase by 2100	Carbon budget of 1460, consistent withend 2 °C average increase by 2100	Carbon budget of 710, consistent with 1.5 °C average increase by 2100	Carbon budget of 710, consistent with 1.5 °C average increase by 2100
R&D strategy	All regions optimally set their R&D Strategy In order to meet their energy demand	All regions optimally set their R&D Strategy in order to achieve the climate targets	All regions achieve the climate targets with no further R&D investments (i.e., R&D investments are equal to the REF scenario)	All regions optimally set their R&D Strategy in order to achieve the climate targets	All regions achieve the climate targets with no further R&D investments (i.e., R&D investments are equal to the REF scenario)
Carbon revenue recycling (in GEM-E3)	Reduction of payroll taxes	Financing of R&D investment, remaining towards the reduction of payroll taxes	Reduction of payroll taxes	Financing of R&D investment, remaining towards the reduction of payroll taxes	Reduction of payroll taxes
Carbon revenue recycling (in WITCH)	Lump sum back to the economy	Lump sum back to the economy	Lump sum back to the economy	Lump sum back to the economy	Lump sum back to the economy

The temperature in REF was Calculated using the MAGICC 6.0 model.

The carbon budgets are the cumulative CO_2_ Emissions counting from the year 2010 to 2100, namely the amount of carbon emission that policy makers’ can still “spend” in order to achieve a given temperature target with a 66% probability. Policy makers can choose to spend the carbon budget differently over time. For instance, if by the year 2050 the carbon budget is already spent, all the emissions from 2050 to 2100 have to be net zero if the given target has to be achieved. In the WITCH model, the carbon budget is achieved by imposing a global carbon tax that is found iteratively until the targeted budget has been reached. In the GEM-E3 model, carbon taxes are endogenously estimated by the model in order to achieve the global emission pathway provided by WITCH.

## Supplementary information

Supplementary Information

## Data Availability

The data generated in this study have been deposited in the zenodo database under accession code DOI 10.5281/zenodo.7755725.

## Code availability

We used the WITCH Integrated Assessment Model along with the MAGICC v6. The MAGICC model can be downloaded in https://magicc.org/. The WITCH model (https://doc.witchmodel.org/) has an open-source version available in https://github.com/witch-team/witchmodel (10.5281/zenodo.7734713). The GEM-E3 model is not available open-source.
