# Supplementary material for: A research and development investment strategy to achieve the Paris climate agreement
Source: Nat Commun. 2023 Jun 16;14:3581. doi: 10.1038/s41467-023-38620-4 (PMC10276053; doi:10.1038/s41467-023-38620-4)
Supplement: Supplementary file 1 — Supplementary Information [file 41467_2023_38620_MOESM1_ESM.pdf]

Supplementary Information

1.WITCH model description

The WITCH (World Induced Technical Change Hybrid) is an integrated assessment model designed to assess climate change mitigation and adaptation policies. It consists of a dynamic global model that integrates in a unified framework the most important elements of climate change. The economy is modelled through an inter-temporal optimal growth model which captures the long term economic growth dynamics. A compact representation of the energy sector is fully integrated (hard linked) with the rest of the economy so that energy investments and resources are chosen optimally, together with the other macroeconomic variables. Land use mitigation options are available through a soft link with a land use and forestry model (GLOBIOM). A climate model (MAGICC) is used to compute the future climate. Climate change impacts the economic output through a damage function, depending also on the rate of investments in adaptation. This allows accounting for the complete dynamic of climate change mitigation and adaptation.

In WITCH, R&D investment is a variable that the model can optimize, the effect of learning-by-research is captured by modelling the knowledge stock, as described in Equation 1. Stocks of knowledge are defined for five technologies, batteries for electric vehicles [1], CCS, solar, wind and advanced biofuels, and overall energy efficiency improvements. At each point in time *t* in region *n*, new ideas are produced using a Cobb-Douglas combination between domestic investments in innovation, I_{rd}, of technology type *rd*, the existing stock of knowledge, RD_{rd}, which captures intertemporal knowledge spillovers, and the knowledge gained from other regions through interregional knowledge spillovers $SPILL_{rd}$. The parameters *a*, *b*, *c*, and *d* are coefficients of the knowledge production function that are sectorally-differentiated, and are calibrated based on [2,3] for all technologies but CCS, solar and wind. For the latter, the parameters are calibrated according to empirical data as shown in supplementary figure 2, supplementary figure 3 and supplementary figure 4.

$$RD_{rd}(t+1,n) = RD_{rd}(t,n)\left(1-\delta_{rd}\right)^{\Delta t} + \Delta t \times a \times I_{rd}(t,n)^{b} \times RD_{rd}(t,n)^{c} \times SPILL_{rd}(t,n)^{d}$$

EQUATION 1

The parameter *a* is a simple scale parameter, while for example the parameter $d = 0.15$ implies that a 1% increase in spillovers increases output of domestic ideas by 0.15%. The return to one's own investment, parameter *b* is slightly higher (b =0.18) for energy efficiency while it contributes the remaining 85% for the other sectors. Only for energy efficiency, the additional knowledge produced depends also on the existing knowledge stock ($c = 0.38 > 0$) since [4] estimates 0.53 for the total effect from knowledge stock without spillovers. Thus deducting the effect from spillovers, we use 0.53-0.15 = 0.38 for the value of c for energy efficiency, while this effect is zero for the other sectors. The knowledge stock is depreciated at δ_{rd} and the spillovers are defined as in Equation 2.

$$SPILL_{rd}(t,n) = \frac{RD_{rd}(t,n)}{\sum_{n\in OECD} RD_{rd}(t,n)} \times \left(\sum_{n\in OECD} RD_{rd}(t,n) - RD_{rd}(t,n)\right)$$

EQUATION 2

The contribution of foreign knowledge through spillovers to the production of new domestic ideas depends on the interaction between two terms: the first describes the absorptive capacity whereas the second captures the distance from the technology frontier, which is represented by the stock of knowledge in OECD countries [2]. The absorptive capacity is defined as the ratio of the region's knowledge stock to the knowledge stock of the frontier regions (OECD in this case). This is based on [5,6] who find that R&D increases the intake of knowledge spillovers. The further one region finds itself from the technological frontier the lesser the country is able to absorb knowledge. This happens because the lack of labs, scientific institutions, and R&D investments in low-income countries creates a barrier to the fruitful utilisation of foreign knowledge. On the other hand, if one country is already at the frontier, it will be less able to profit from foreign knowledge.

The knowledge stock for energy efficiency is combined with energy supply and autonomous energy efficiency improvements to form energy services. Energy services are then used as an input in production of the final good. Conversely, for the other technologies the knowledge stock is used to lower installation costs, SC, which are determined by a two-factor learning curve as in Equation 3.

$$\frac{SC_j(t,n)}{SC_j(0,n)} = \left(\frac{RD_j(t,n)}{RD_j(0,n)}\right)^{-lbr_{factor}} \left(\frac{wcum_j(t,n)}{wcum_j(0,n)}\right)^{-lbd_{factor}}$$

Equation 3

In two-factor learning curves (Equation 3) investment costs decrease as a result of the accumulation of knowledge (learning-by-researching) or experience (learning-by-doing), which are independent. The accumulation of knowledge is generated by investments in research and development, as discussed above, while the stock of experience is modelled through global cumulative installed capacity, *wcum* (full global technology spillover is assumed). In Equation 3 lbr_{factor} and lbd_{factor} measure the strength of the learning effect independently. The former measures the effect of the knowledge stock gained via Equation 2; the latter measures the effect of the cumulative capacity on the reduction of installation costs. They relate to the corresponding learning rates, lbr_{rate} and lbd_{rate}, which measure the rate at which unit costs decrease for each doubling of the knowledge or capacity stock, through the following relationship: $lbd_{rate} = 1\text{-}2^{lbd}$. The same applies to learning-by-researching.

1.1 Calibration of the knowledge stock for the newly added technologies in WITCH

The Solar, Wind and CCS knowledge stock calibration was undertaken using patent data. Data on patents is sourced from the Statistics of the OECD - Environmental Statistics - Innovation in environment-related technologies - Patents - Technology development (downloaded March 2019). OECD patent statistics presented here are constructed using algorithms developed by the OECD Environment Directorate drawing on data extracted from the OECD STI Micro-data Lab: Intellectual Property Database, http://oe.cd/ipstats. Consistent with other patent statistics provided in OECD.Stat, only published applications for "patents of invention" are considered (i.e. excluding utility models, petty patents, etc.). The relevant patent documents are identified using search strategies for environment-related technologies (ENV-TECH) which were developed specifically for this purpose. They allow identifying technologies relevant to environmental management and climate change mitigation. From the OECD patent statistics, we select indicators of patents with family size greater than two, indicating patents which are protected in at least two jurisdictions, to account for patent quality [7]. Patent statistics are then used to calculate knowledge stocks, using the perpetual inventory method [8,9] with an assumed discount rate of 10 percent. Patent data was then linked with information on energy RD&D budgets from the IEA [10] in order to calculate the average global value of an energy patent (by technology). Lastly, patent knowledge stocks were multiplied by calculated global patent value in order to translate knowledge stocks values into monetary values and to thus match the units of measurement used in the WITCH model.

Once the historical knowledge stock has been built, we calibrate Equation 1. Firstly, the resulting historical $RD_{rd}(t,n)$ is used in equation 2 to calculate the SPILL_{rd} variable. Secondly, both SPILL_{rd} and $RD_{rd}(t,n)$

are used to calibrate equation 1 the $RD_{rd}(t, n)$. This means that the parameters of equation 1 for the *rd* technologies were calibrated using historical data. We use a genetic algorithm [11] to find parameters a, b, c, d. In the case of these three newly added technologies, we assume that a=1 and c=0 as the other technologies in WITCH with the exception of energy efficiency. However, unlike other technologies in WITCH, here we assume that the parameters vary regionally. The resulting parameters are shown in supplementary figure 1. Genetic algorithms are useful heuristic methods for optimization, in this case we want to find the parameters b and d that better fit regional historical knowledge stocks, that is the ones that minimise the errors (square root of sum of squares between predicted and observed) between the historical data and the fitted data. In the method used [11] the parameters of the knowledge stock equation, b and d are transformed at each 'generation' (i.e. iteration) resulting in a 'population' that is more likely to minimise the errors between the observed and the predicted knowledge stock. The new set of parameters are generated by disturbing the previous ones. The trial mutation of parameters are evaluated and if their objective function is lower than the population keeps that mutation. The algorithm stops when the objective function is lower than a certain threshold or a maximum number of population has been achieved. Here we used 100 as the maximum population number and -Inf as the objective function threshold. Supplementary Figures 2 to 4 show the goodness of fit found for all the newly added technologies per region.

For some technologies, the spillover parameter d is close to zero. This is the case of first movers that are very innovative, such as OECD regions. This may also happen in countries that have historically extremely low R&D knowledge stock as they do not innovate enough on their own to benefit from spillovers. This assumption relies on the historical patent data provided by OECD. Based on equation 2 for the calculation of spillovers, a spillover parameter close to zero implies that foreign knowledge has no effect on a region's innovation as compared to own investments in R&D. In other words, it assumes that historically, these regions have not relied significantly on foreign knowledge from OECD regions to build their knowledge stock. We acknowledge that this assumption may be somewhat normative given that they are based on the way the spillover parameter is calculated [2]. Thus we provide sensitivity results on the choice of this parameter.

The resulting regional calibration fitting has been evaluated against the regional knowledge stock built from the OECD patent and IEA database, and it is presented in supplementary figure 2, supplementary figure 3, supplementary figure 4, for CCS, wind and Solar technologies respectively. The parameters for these three technologies are presented in supplementary figure 1. Here we assume that R&D investments can have increasing returns to scale. The regions that have higher b value are the regions whose knowledge stock depends highly on their own R&D investments, whereas high d parameters suggest a high dependency of the foreign knowledge (knowledge spillovers). Supplementary Figures 2-4 show, generally, a good agreement of the fitted parameters to equation 1.

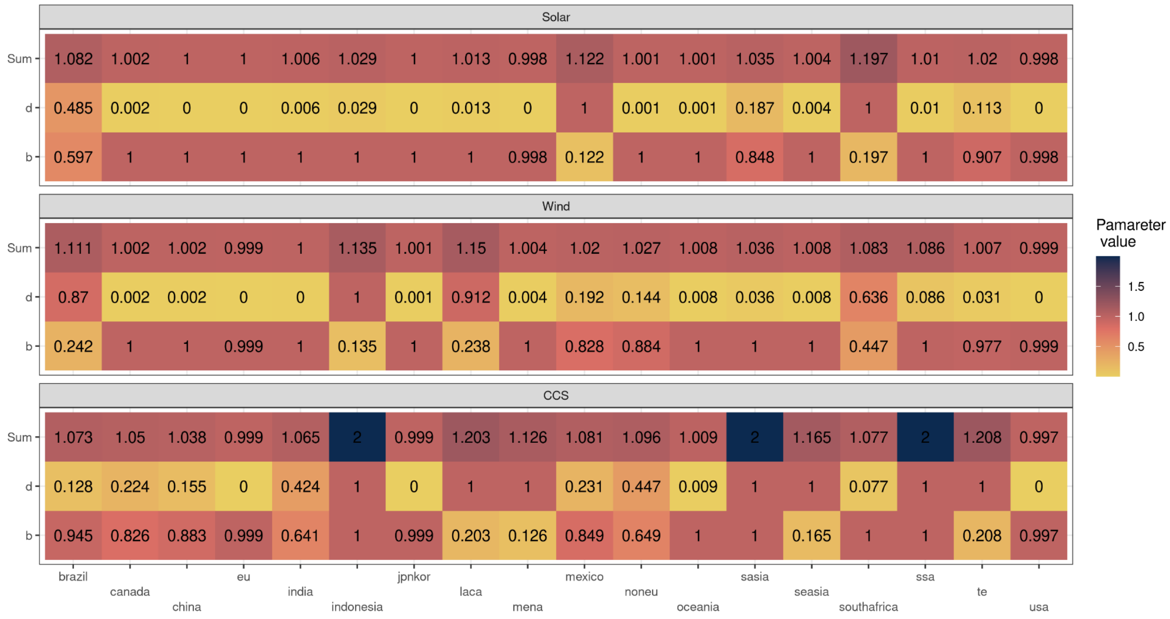

Supplementary figure 1 Regional calibration parameters of Equation 1 rounded to 3 digits, for solar, Wind and CCS. The regional definition is presented in supplementary Table 3. the b parameter refers to the regional elasticity of regional investment on the knowledge stock and the parameter d refers to the spillovers.

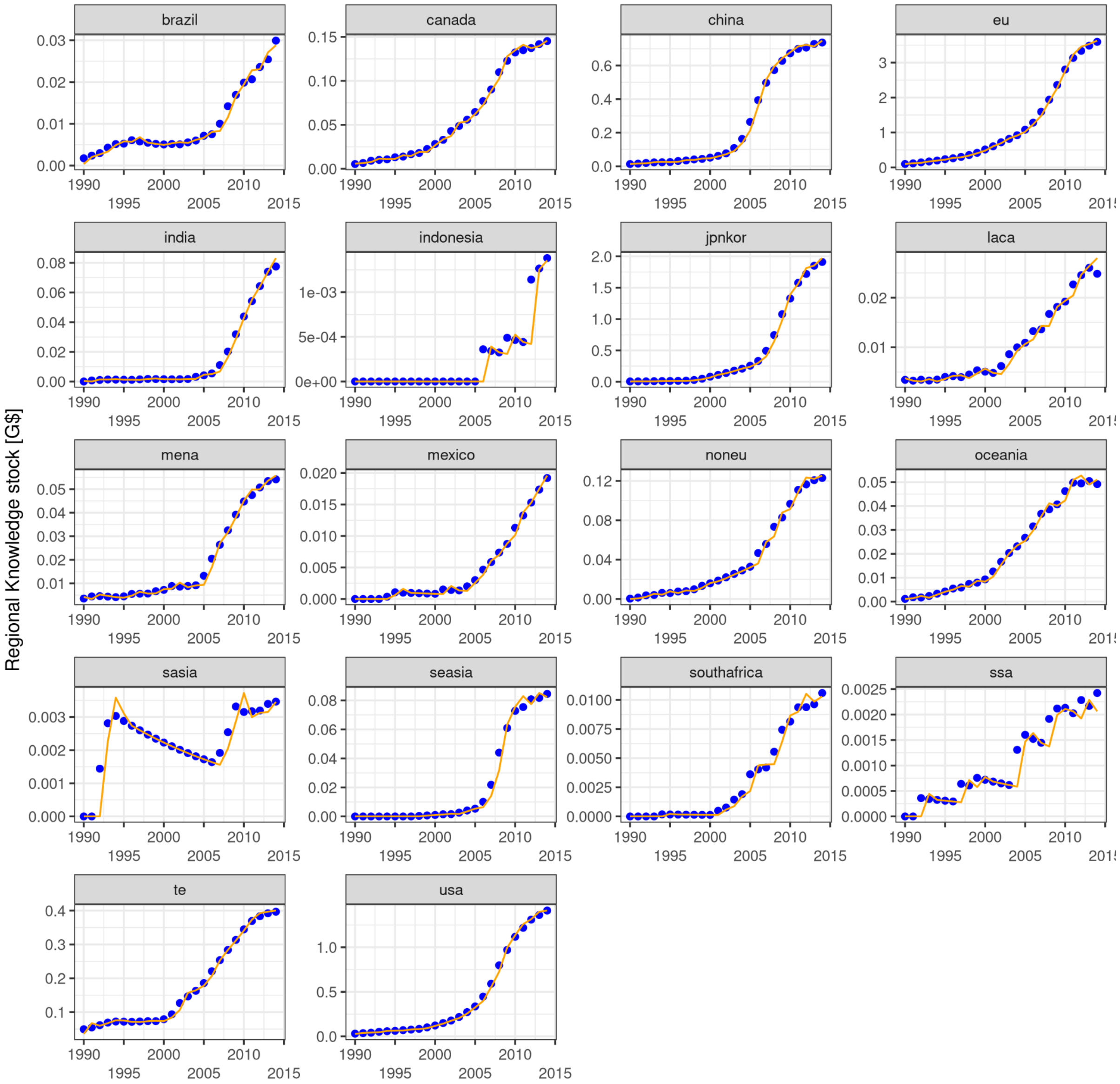

SUPPLEMENTARY FIGURE 2 REGIONAL WIND KNOWLEDGE STOCK CALIBRATION IN THE WITCH MODEL. THE ORANGE LINE REPRESENTS THE FITTED KNOWLEDGE STOCK ACCORDING TO EQUATION (1) AND THE BLUE DOTS REPRESENT THE REGIONAL DATA BASED ON OECD PATENT DATA AND IEA R&D INVESTMENT DATA FOR THE AVAILABLE YEARS IN THE DATABASE. THE REGIONAL DEFINITION IS PRESENTED IN SUPPLEMENTARY TABLE 3.

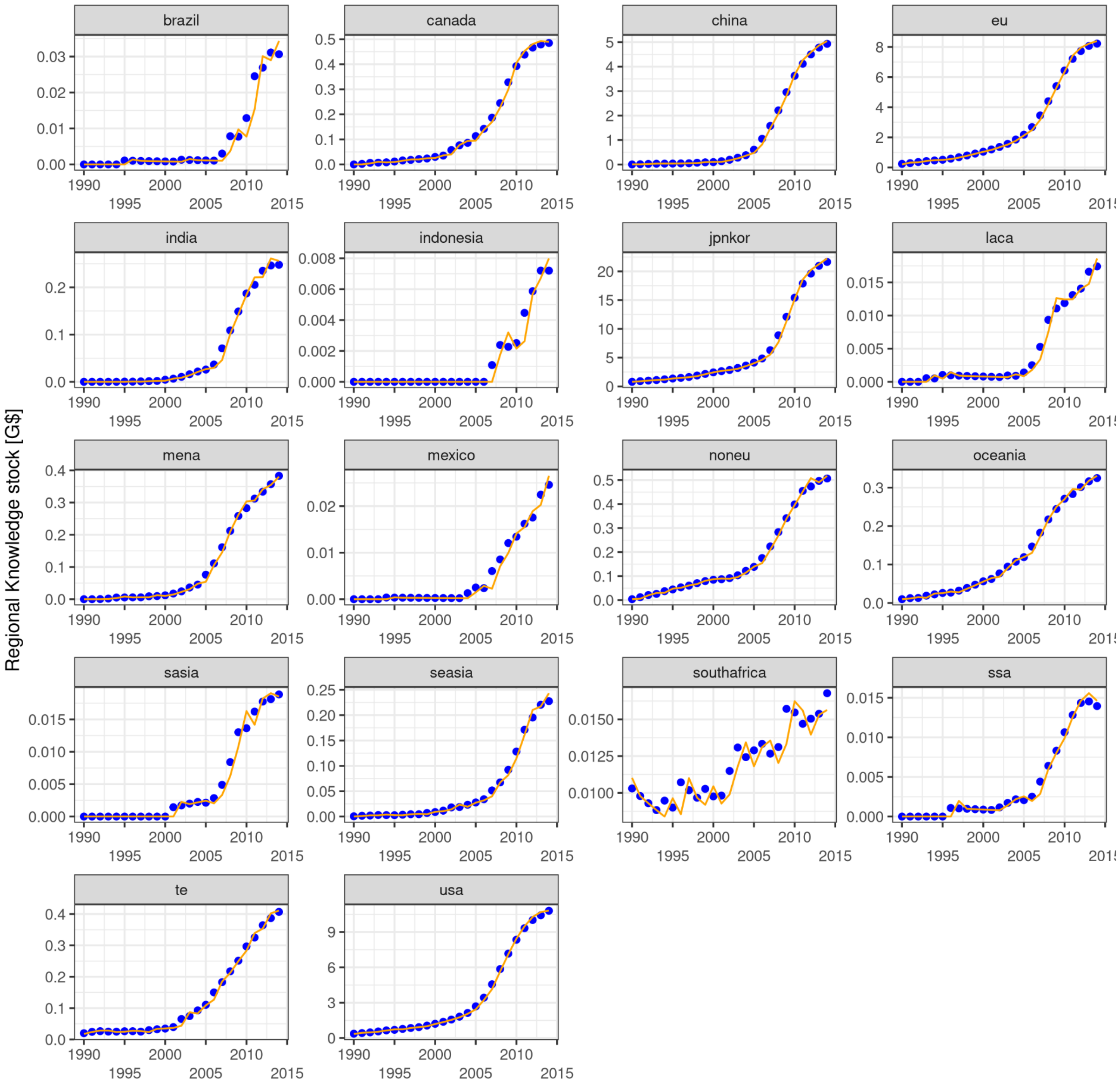

SUPPLEMENTARY FIGURE 3 REGIONAL SOLAR KNOWLEDGE STOCK CALIBRATION IN THE WITCH MODEL. THE ORANGE LINE REPRESENTS THE FITTED KNOWLEDGE STOCK ACCORDING TO EQUATION (1) AND THE BLUE DOTS REPRESENT THE REGIONAL DATA BASED ON OECD PATENT DATA AND IEA R&D INVESTMENT DATA FOR THE AVAILABLE YEARS IN THE DATABASE. THE REGIONAL DEFINITION IS PRESENTED IN SUPPLEMENTARY TABLE 3.

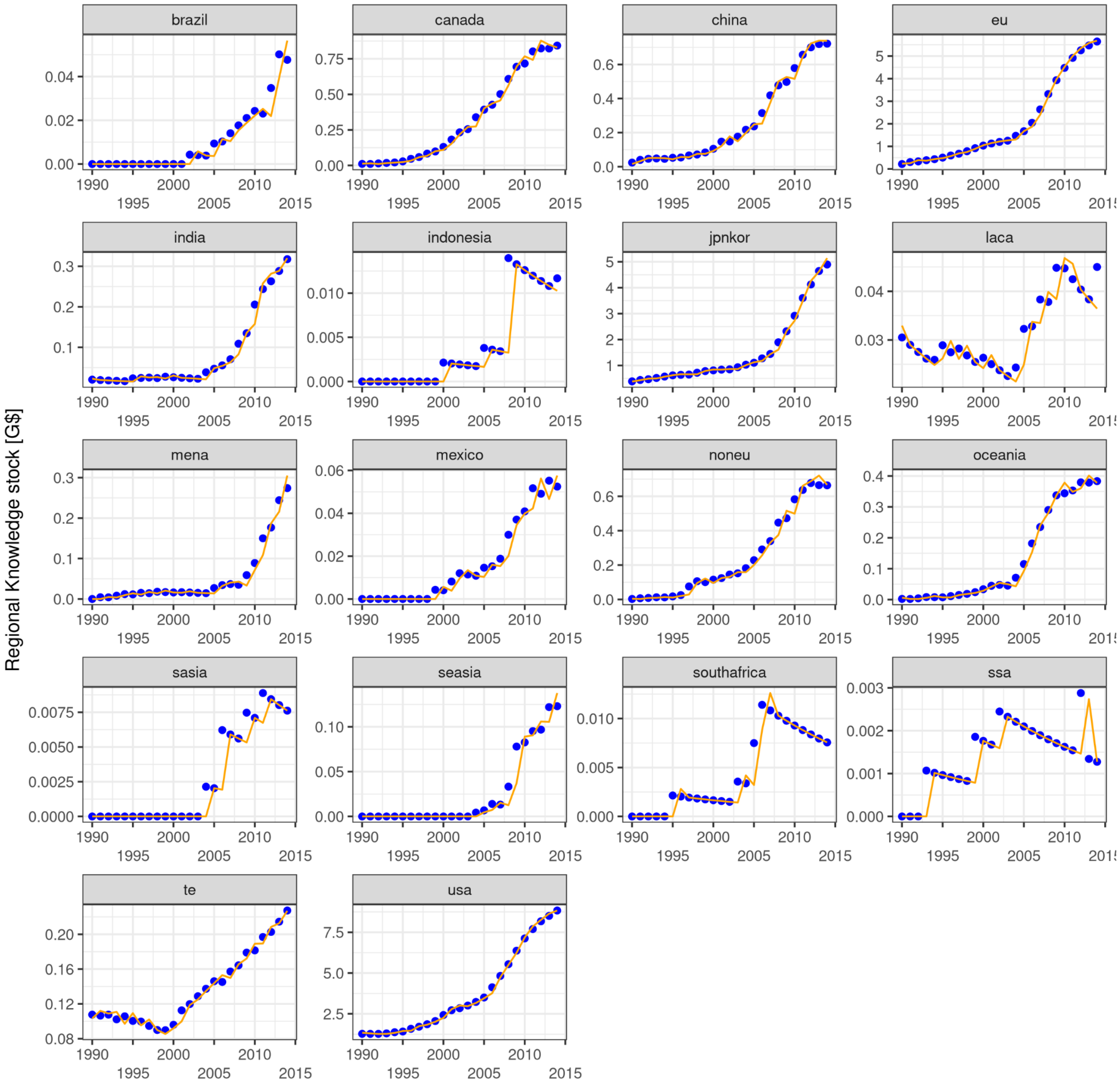

Supplementary figure 4 Regional CCS knowledge stock calibration in the WITCH model. The orange line represents the fitted knowledge stock according to equation (1) and the blue dots represent the regional data based on OECD patent data and IEA R&D investment data for the available years in the database. The regional definition is presented in Supplementary Table 3.

Supplementary Table 3: WITCH model regional definition

WITCH regions	Description
BRAZIL	Brazil
CANADA	Canada
CHINA	China
EU	European union
NONEU	non EU Europe
INDIA	India
INDONESIA	Indonesia
JPNKOR	Japan and South Korea
LACA	Latin America and Caraibes (except Brazil and Mexico)
MENA	Middle-east and North Africa
MEXICO	Mexico
OCEANIA	Oceania
SASIA	South Asia
SEASIA	South-East Asia
SOUTHAFRICA	South Africa
SSA	Sub-Saharian African (except South Africa)
TE	Transition Economies (including Russia)
USA	United States of America

Supplementary figure 5 shows the results of the sensitivity to the spillover parameter d of equation 1 (supplementary figure 1). Several sensitivity scenarios are presented, where the parameter d is changed to 0.01, 0.05, 0.1 and 0.15 for the regions where the calibration has yield $d<0.01$, $d<0.05$, $d<0.1$ and $d<0.15$, respectively. Overall, when the spillover parameter is higher, less own R&D investment is needed because the knowledge stock is increased through the absorption of foreign knowledge. This mostly affects the regions of Europe, USA and Japan and South Korea, which are less likely to be substantially dependent on knowledge produced in non-OECD countries.

Looking at the effects per technology, CCS results are not very sensitive to the calibration of the spillover parameter. For solar, a higher spillover parameter results in lower total need for R&D investment. Importantly, in the WITCH model, the floor cost for solar is achieved very rapidly, and the learning-by-doing rates are very high. This leads to lower sensitivity to the knowledge stock variable. Wind is the technology with sensitivity to the spillover parameter. While this changes the magnitude of the investments, it does not change the relationship between the scenarios. In this case, a higher spillover parameter displaces R&D investments to the future, when such investments are less costly.

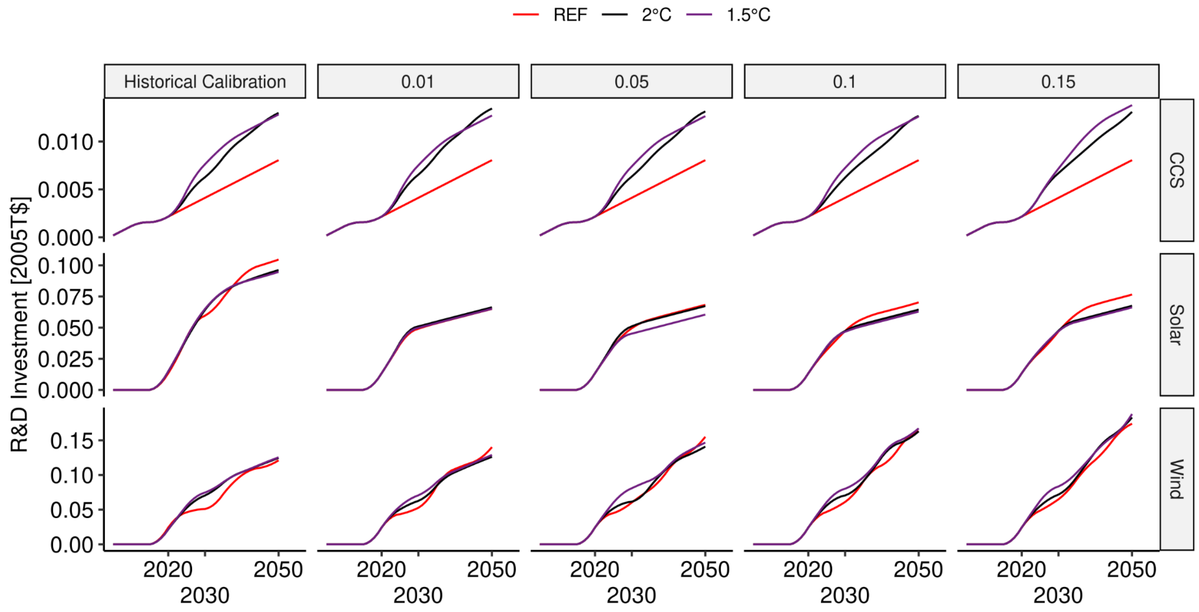

Supplementary figure 5 Global R&D investment sensitivity to the spillover parameter ('d') of equation 1, in the WITCH model. The 'historical calibration' refers to the results of the genetic algorithm calibration to historical data.

2.GEM-E3 model description

The GEM-E3 model is a hybrid, large scale general equilibrium model that provides insights on the macroeconomic and sectoral impacts of the interactions of the environment, the economy, and the energy system. The model simultaneously simulates the equilibrium of multiple markets, including the labour and capital markets as well as several markets for aggregate goods and services. The model optimizes firm and household behaviour by ensuring maximum welfare for households and minimum production cost for firms. The model is calibrated to the latest statistics (GTAP 10, IEA, UN, ILO) while Eurostat statistics have been used for the EU model. The model is recursive dynamic with myopic agents, while the dynamic feature is driven by the accumulation of capital with a time horizon to 2100. GEM-E3 features endogenous bilateral trade following the Armington assumption on preferences between domestic and imported goods. GEM-E3 model features an imperfect labour market and can thus estimate the policy impacts on involuntary unemployment levels. The model is highly detailed in terms of sectoral and regional disaggregation, featuring 46 regions (EU28 Member States and G20 countries) and 67 categories of economic activities, including a separate representation of the (10) sectors that produce low-carbon power supply technologies (e.g. PV equipment), electric cars and batteries, and advanced household appliances. The model also includes a detailed representation of the power supply system (12 power technologies) and a detailed transport module including private and public transport modes). The GEM-E3 environment module covers all GHG emissions and a wide range of abatement options, as well as a thoroughly designed carbon market structure (e.g., grandfathering, auctioning, alternative recycling mechanisms).

GEM-E3 model features endogenous technical progress as described in [12] and further developed by E3Modelling (https://e3modelling.com/). The model identifies both public and private R&D, each having different characteristics in terms of funding and knowledge diffusion. Learning is the result of research efforts (Learning by Research, LbR) and the result of gained experience (Learning by Doing, LbD). LbD in GEM-E3 indicates that costs are reduced with the doubling of capacity, however, agents are myopic and

not aware of this effect. The cost reduction occurs once their investment decision is made. The gains from the learning effect occur with a one period lag.

LbR investments by firms and government create a stock through a motion equation shown in Equation 4. Accumulation of past innovations results in diminishing returns to scale (fishing-out effect) through δ_{RD}.

$$CUMRD_{j,r,t} = (1-\delta_{RD})^{\Delta t} \cdot CUMRD_{j,r,t-1} + RD_{pr,r,t-1} \cdot \left[\frac{(1-\delta_{RD})^{\Delta t+1}-1}{(1-\delta_{RD})-1}\right]$$

EQUATION 4

Further to LbD and LbR, the model also simulates knowledge diffusion by identifying four types of spillovers, namely own-sector spillovers, direct cross-sectoral spillovers, indirect cross-sectoral spillovers and interregional own-sector spillovers. The level of direct diffusion depends on the spillover matrices that are constructed with the patent/citation approach, i.e. forward citations indicate the flow of knowledge, and can be found in [13]. The level of indirect diffusion depends on the demand for intermediate goods as the knowledge created in one industry facilitates the processes in other industries that use its goods as production inputs. It should be noted that current modelling of spillovers does not include impacts of patents with extreme value.

The GEM-E3 R&D module has been further developed so as to include human capital and endogenous skill supply which then directly impact the absorptive capacity of the economy [14]. As a result, knowledge absorption capacity depends on skill availability and human capital. Overall, the total factor productivity (TFP) of a sector is estimated by Equation 5, Equation 6, Equation 7 and Equation 8. where CUMRD is cumulative private R&D, CUMPBRD is cumulative public R&D, SPILLM is the spillovers matrix, HC the human capital stock, β the learning rates that depend on the human capital stock and γ the absorption rates. The RDBASE is an exogenous parameter that is used to set the minimum private RD investments that is required in order to start getting productivity by the RD investments.

$$TFP_{j,r,t} = TFP(Private)_{j,r,t} \cdot TFP(Public)_{j,r,t} \cdot TFP(Spillovers)_{j,r,t}$$

EQUATION 5

$$TFP(Private)_{j,r,t} = \left(\frac{max\{CUMRD_{j,r,t};RDBASE_j\}}{max\{CUMRD_{j,r,0};RDBASE_j\}}\right)^{\beta^1_{r,t}}$$

EQUATION 6

$$TFP(Public)_{j,r,t} = \left(\frac{\Sigma_r CUMPBRD_{j,r,t}}{\Sigma_r CUMPBRD_{j,r,0}}\right)^{\beta^2_{r,t}}$$

EQUATION 7

$$TFP(Spillovers)_{j,r,t} = \left(\frac{max\{\Sigma_{i,c,} SPILLM_{i,j,r,c,t} \cdot CUMRD_{i,c,t}; \Sigma_{i,c} SPILLM_{i,j,r,c,t} \cdot RDBASE_i\}}{max\{\Sigma_{i,c,} SPILLM_{i,j,r,c,0} \cdot CUMRD_{i,c,0}; \Sigma_{i,c} SPILLM_{i,j,r,c,0} \cdot RDBASE_i\}}\right)^{\beta^3_{r,t}}$$

EQUATION 8

where:

$$\beta^1_{r,t} = \beta^1_{r,0}\left(\frac{HC_{r,t}}{HC_{r,0}}\right)^{\gamma_1}$$

$$\beta^2_{r,t} = \beta^2_{r,0}\left(\frac{HC_{r,t}}{HC_{r,0}}\right)^{\gamma_2}$$

$$\beta^3_{r,t} = \beta^3_{r,0}\left(\frac{HC_{r,t}}{HC_{r,0}}\right)^{\gamma_3}$$

Typically, in the GEM-E3 model public R&D investment contribute to the global knowledge stock that is available to all regions simultaneously, while private R&D investment creates knowledge that is available to the investing firm/industry and can only be diffused to the rest of the regions through spillover matrices and with a one period delay (i.e. 5 years). However, in order to partly harmonize the methodological approaches across the two models for the purposes of the current analysis, all R&D investment that is introduced as an input from the WITCH to the GEM-E3 model, is considered to be state-funded but with limited diffusion to other regions that is subject to the spillover matrices, similar to the standard approach for private R&D investments. In this analysis, private R&D investments in GEM-E3 are kept constant to the Reference scenario levels.

2.1Regional allocation of R&D investments (From WITCH to GEM-E3)

The spatial resolution of both models is not fully compatible. Therefore, a reallocation of the optimal R&D investments calculated by the WITCH model has been carried out. Downscaling our analysis on an EU-MS level in the GEM-E3 model requires a disaggregation of EU-wide R&D investment estimated by the WITCH model. We introduce the optimal EU R&D investment from the WITCH model to the GEM-E3 model by taking stock of regional and country-level R&D data and existing literature. In particular, the IEA database (http://wds.iea.org/WDS/TableViewer/dimView.aspx?ReportId=1399) on "Detailed Country R&D Budgets" has been consulted for all 3 technologies assessed, namely advanced biofuels, CCS and batteries for vehicles. However, the database is incomplete as it does not include data for all EU-MS and data points are missing for a number of reporting countries and years. We have thus employed additional data sources for batteries [15,16], CCS [17] and advanced biofuels [UNCTAD 2015]. The shares of each EU-MS in total EU28 R&D investment are presented in Supplementary Table 4 below.

Supplementary Table 4 EU-MS shares in total EU28 R&D investment assumed in the GEM-E3 model for the incorporation of EU-wide R&D investment from the WITCH model.

Country	CCS	Vehicle Batteries	Advanced Biofuels
Austria	1.8%	6.4%	3.2%
Belgium	1.2%	1.7%	1.7%
Bulgaria	0.2%	0.2%	0.2%
Cyprus	0.2%	0.2%	0.2%
Croatia	0.2%	0.2%	0.2%
Czech Rep.	0.5%	4.0%	0.6%

Germany	17.7%	3.6%	11.0%
Denmark	0.2%	0.2%	7.1%
Spain	4.3%	0.2%	3.1%
Estonia	0.2%	0.2%	0.2%
Finland	0.2%	0.4%	8.3%
France	31.2%	38.7%	25.8%
UK	19.7%	12.5%	7.7%
Greece	0.2%	0.2%	0.2%
Hungary	0.2%	6.7%	0.2%
Ireland	0.2%	0.2%	3.2%
Italy	4.4%	0.2%	5.3%
Lithuania	0.2%	0.2%	0.2%
Luxemburg	0.2%	0.2%	0.2%
Latvia	0.2%	0.2%	0.2%
Malta	0.2%	0.2%	0.2%
Netherlands	11.7%	0.2%	9.8%
Poland	3.2%	19.8%	2.8%
Portugal	0.2%	0.2%	0.6%
Slovakia	0.2%	0.2%	0.5%
Slovenia	0.2%	0.2%	0.5%
Sweden	0.2%	2.0%	6.2%
Romania	0.2%	0.2%	0.2%

3.Two –factor learning

IAMs representing the energy system may include representations of induced technical change either endogenously or exogenously [18,19,20]. These representations mimic the technology cost reductions that may occur due to learning, most frequently learning-by-doing and learning-by-researching.

The empirical literature that studies the evolution of cost of energy technologies informs the model assumptions that drive ITC. Some approaches/models use historical data to support the assumption that costs decrease as a function of cumulative capacity or over time [21]. Others rely on expert elicitations of how future costs may unfold [22].

In IAMs that model ITC endogenously, the costs are modelled through learning curves, also called experience curves. In this case, costs are reduced as a function of cumulative installed capacity (learning-by-doing) [23], or investments in Research and Development (R&D) (i.e.

learning-by-researching) or even other factors such as for example Economy of scale or learning-by-interacting [24].

The application of two-factor learning curves to model technology costs may be constrained by the availability of information on energy R&D investments at the country level [22]. The GEM-E3 [13] includes spillover matrices to account for bilateral spillovers and compute learning rates that depend on the human capital stock and the regional and/or sectoral absorption rates [25].

Supplementary figure 6 shows the relation between the two components of the two factor learning curve. Costs are high when the installed capacity is low, and therefore R&D investments are high in order to lower the costs that will allow higher deployment rates.

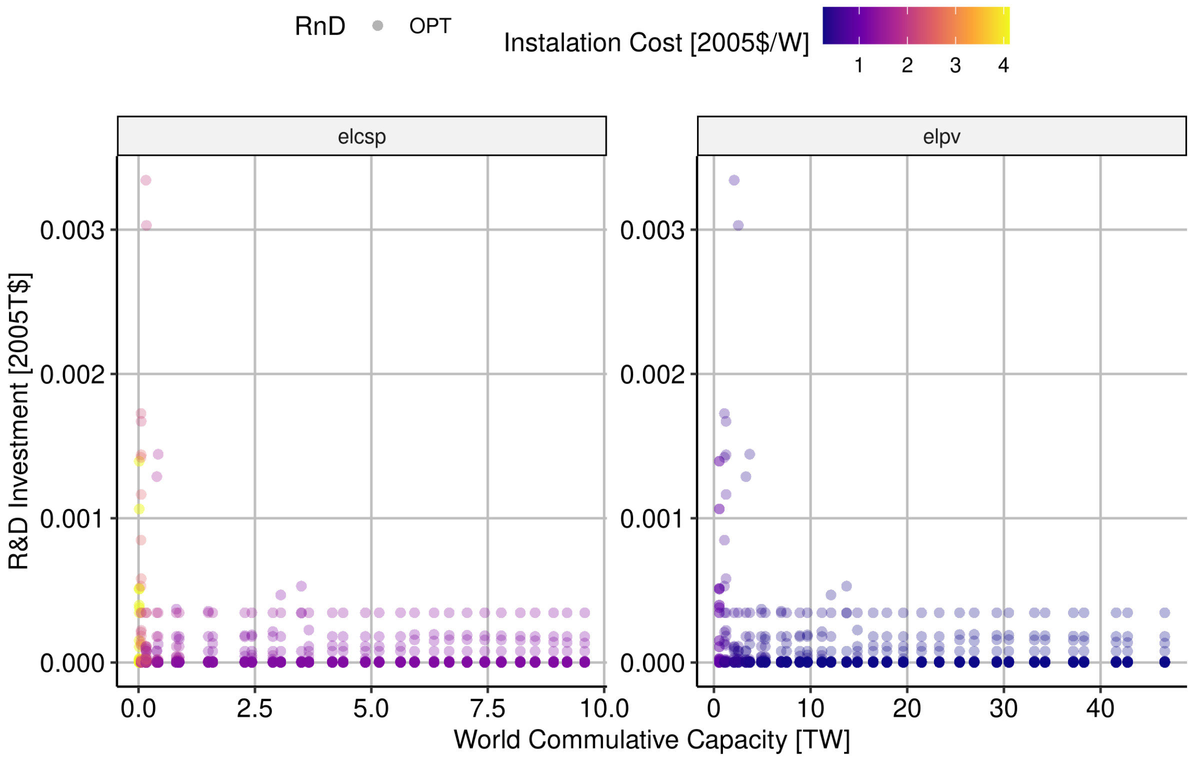

Supplementary figure 6 The effect of the two-factor-learning curve. R&D investments as a function of world capacity for the solar technologies. Each point represents the values of a given region in a given year. The colour scale represents the installation costs. elcsp=concentrated solar power and elpv=photovoltaic.

4.Sensitivity to the learning rates

The learning rates shown in Table 1 are based on literature, however they constitute a normative choice that may have an effect on the results. Here we present the results of our sensitivity analysis. Supplementary figure 7 presents the sensitivities of the global cumulative R&D investments of each technology due to the perturbations in each of the technologies learning-by-research rates. The results show that technologies are especially sensitive to their own learning rate. Nevertheless, except advanced biofuels (especially in the carbon budget of 710 scenario) and wind technologies, technologies do not show substantial variations. The perturbations applied here are of +-50%, which are extreme cases of learning rate change. The largest changes happen when perturbing the rates of all the technologies at the same time (supplementary figure 8), which is a very unlikely scenario.

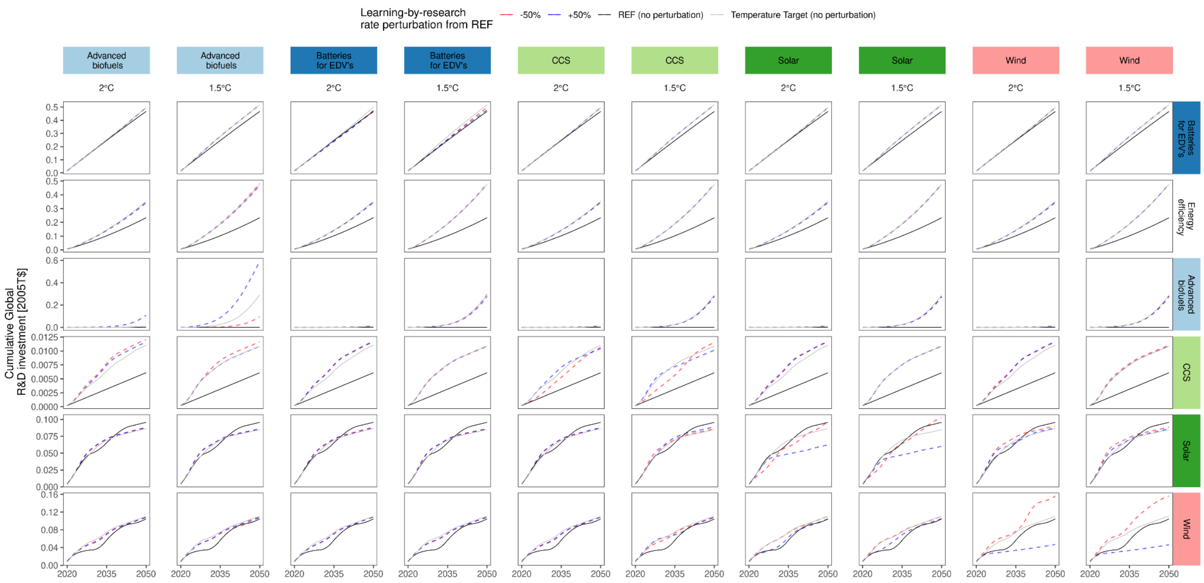

Supplementary figure 7 Sensitivity of the global cumulative R&D to changes is each technologies' learning-by-research rates of the selected R&D technologies.

Advanced biofuels are one of the technologies that shows higher sensitivity. Supplementary figure 8 shows the sensitivity of advanced biofuels when changing both the learning-by-doing and the learning-by-research rate individually and simultaneously. It shows that advanced biofuels learning rate changes may have a small effect on CCS and energy efficiency R&D investment. Moreover, the R&D investment in advanced biofuels is more sensitive to the learning-by-research rate changes than the learning-by-doing ones.

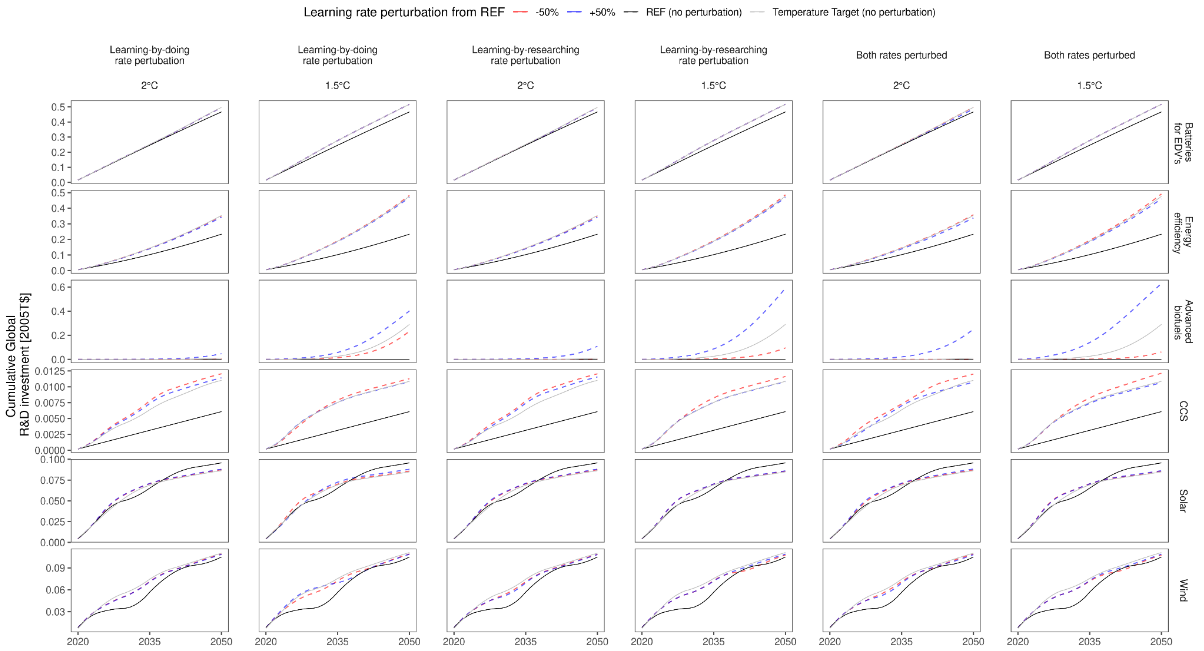

Supplementary figure 8 Sensitivity of the global cumulative R&D to changes in advanced biofuels learning rates on the selected R&D technologies.

Finally, we have analysed a setting where all the technologies' learning rates are perturbed at the same time, shown in supplementary figure 9. In this case we find once more that wind and advanced biofuels are the most sensitive technologies. Concerning wind, and to some extent also solar, this result is due to the

fact that the leaning-by-research rates found in [26] (as in Table 1) is very high for wind while the learning-by-doing rates are relatively low, therefore a +-50% perturbation on the learning-by-research creates a large impact.

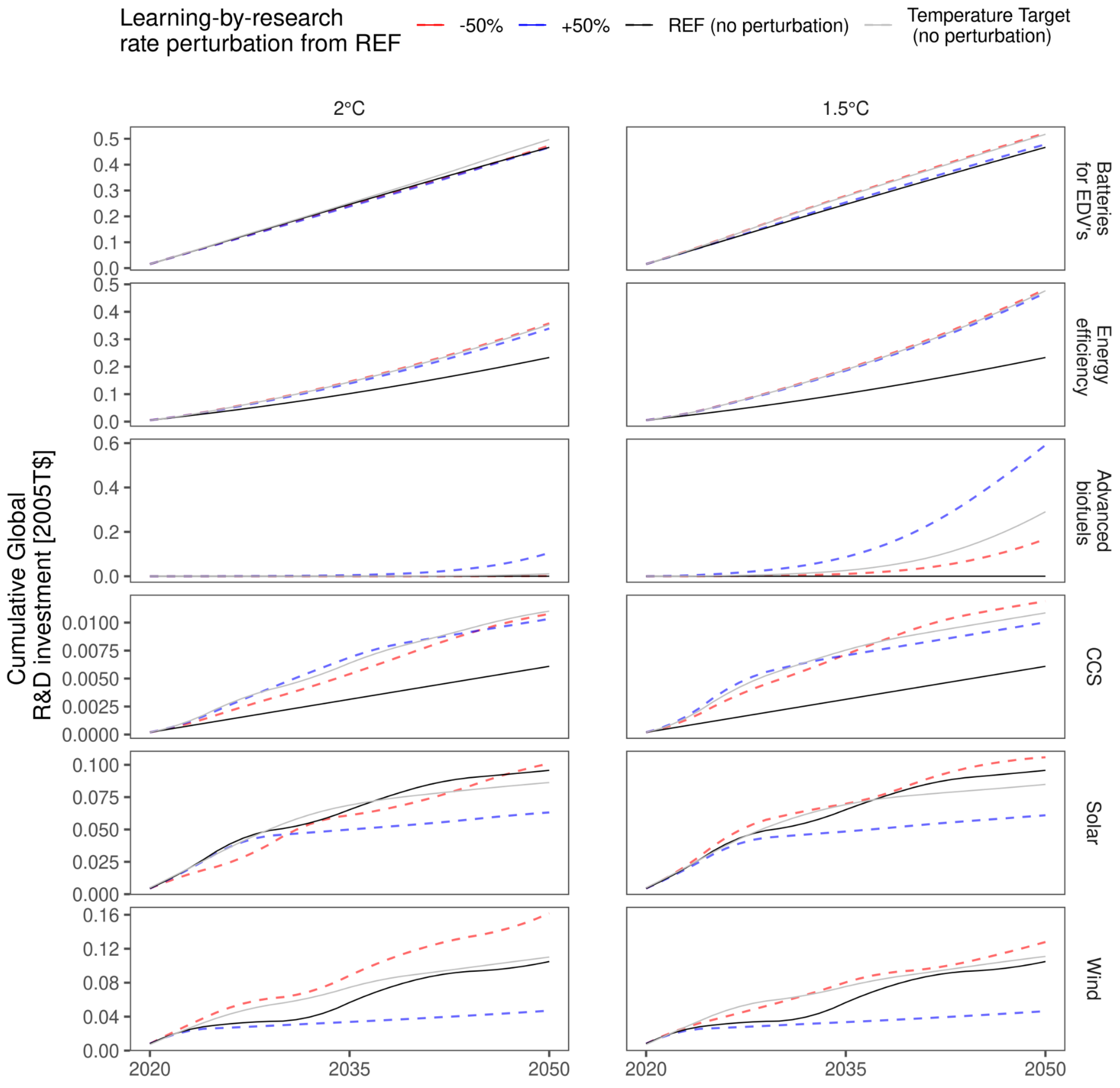

Supplementary figure 9 Sensitivity of the global cumulative R&D to changes in the learning rates of all technologies simultaneously on the selected R&D technologies. Please note that for the 1.5°C scenario the -50% change in learning by research rate was not feasible therefore we show the results for -80%.

5.Regional cumulative R&D investment per technology

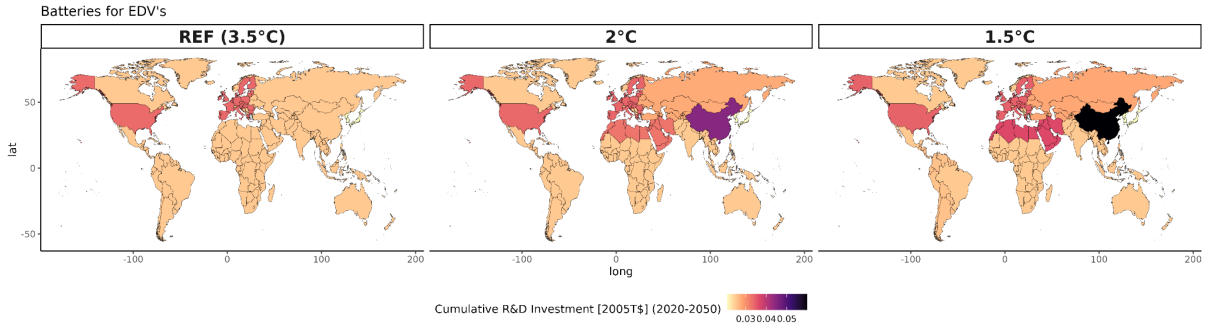

SUPPLEMENTARY FIGURE 10 CUMULATIVE R&D INVESTMENT IN BATTERIES FOR EDV'S FROM 2020 TO 2050. SOURCE: WITCH MODEL. MADE WITH NATURAL EARTH.

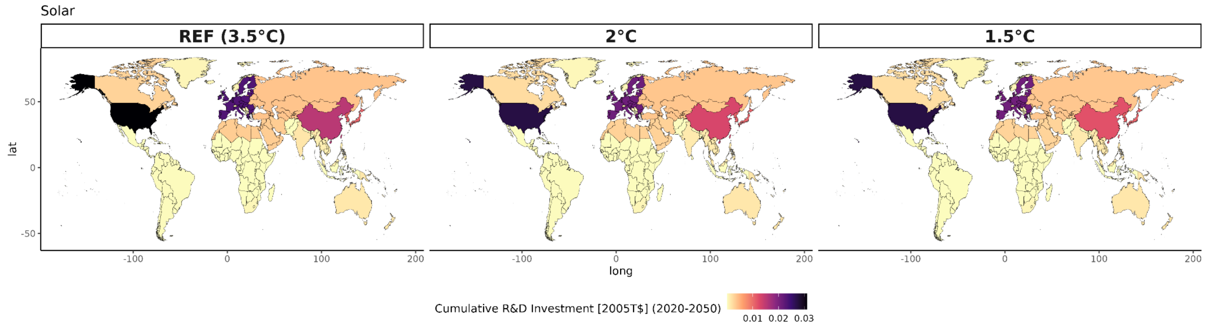

SUPPLEMENTARY FIGURE 11 CUMULATIVE R&D INVESTMENT IN SOLAR TECHNOLOGIES FROM 2020 TO 2050. SOURCE: WITCH MODEL. MADE WITH NATURAL EARTH.

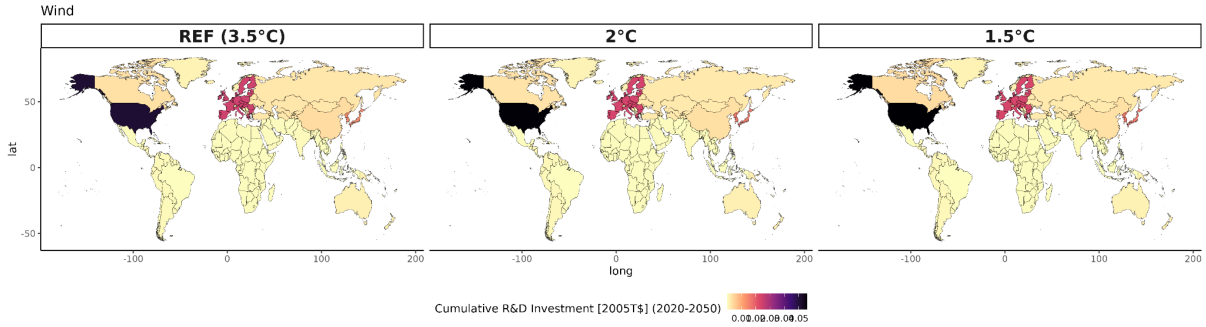

SUPPLEMENTARY FIGURE 12 CUMULATIVE R&D INVESTMENT IN WIND TECHNOLOGIES FROM 2020 TO 2050. SOURCE: WITCH MODEL. MADE WITH NATURAL EARTH.

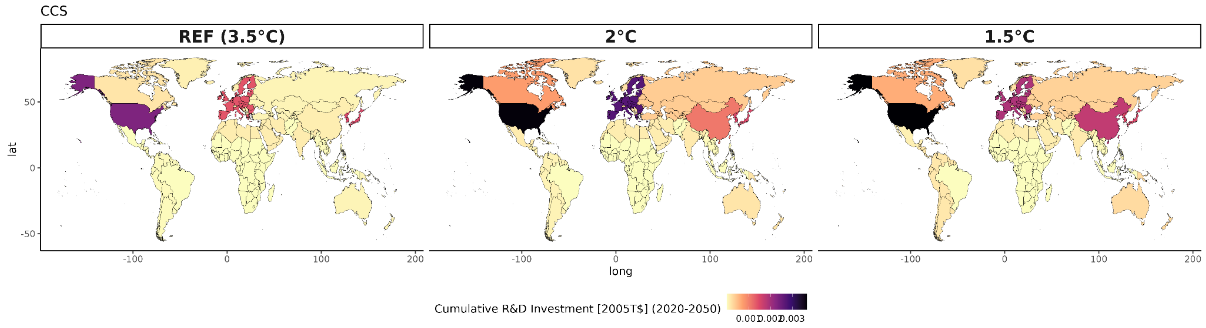

SUPPLEMENTARY FIGURE 13 CUMULATIVE R&D INVESTMENT IN CCS TECHNOLOGIES FROM 2020 TO 2050. SOURCE: WITCH MODEL. MADE WITH NATURAL EARTH.

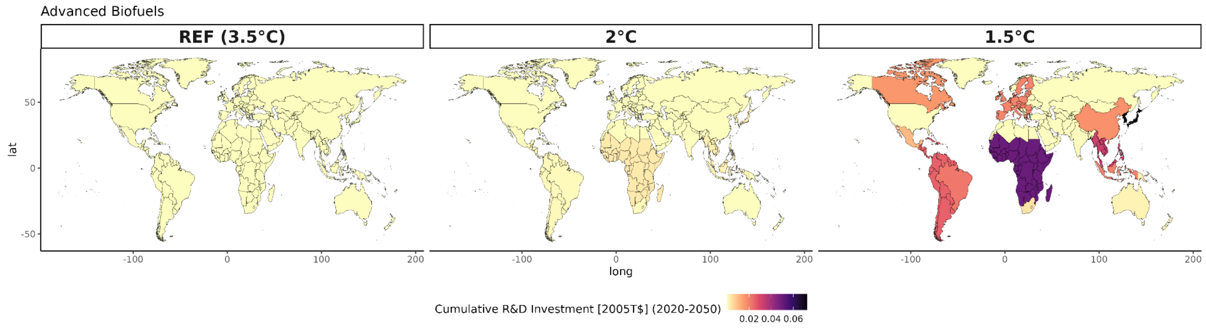

SUPPLEMENTARY FIGURE 14 CUMULATIVE R&D INVESTMENT IN ADVANCED BIOFUELS FROM 2020 TO 2050. SOURCE: WITCH MODEL. MADE WITH NATURAL EARTH.

6.R&D investment as a share of GDP

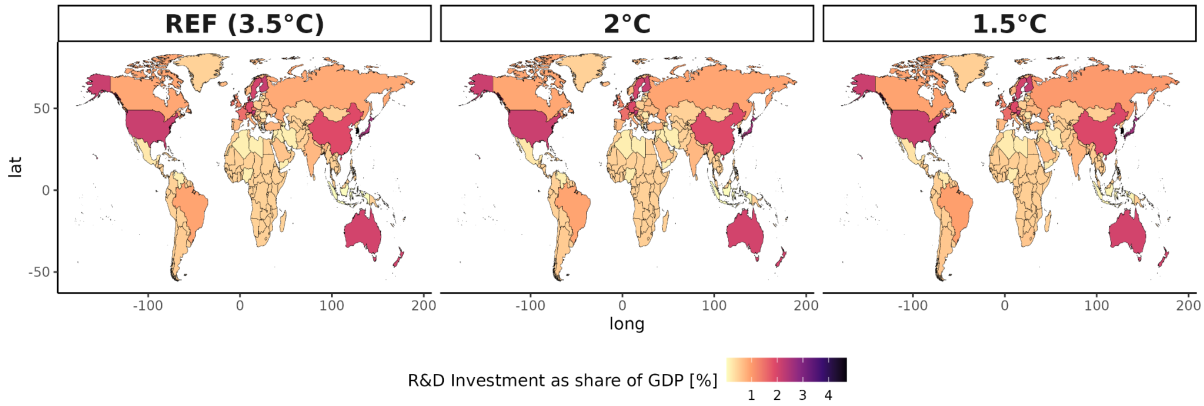

SUPPLEMENTARY FIGURE 15 R&D INVESTMENT AS A SHARE OF GDP IN 2050. SOURCE: GEM-E3 MODEL. MADE WITH NATURAL EARTH.

7.R&D investment as a share of carbon revenues

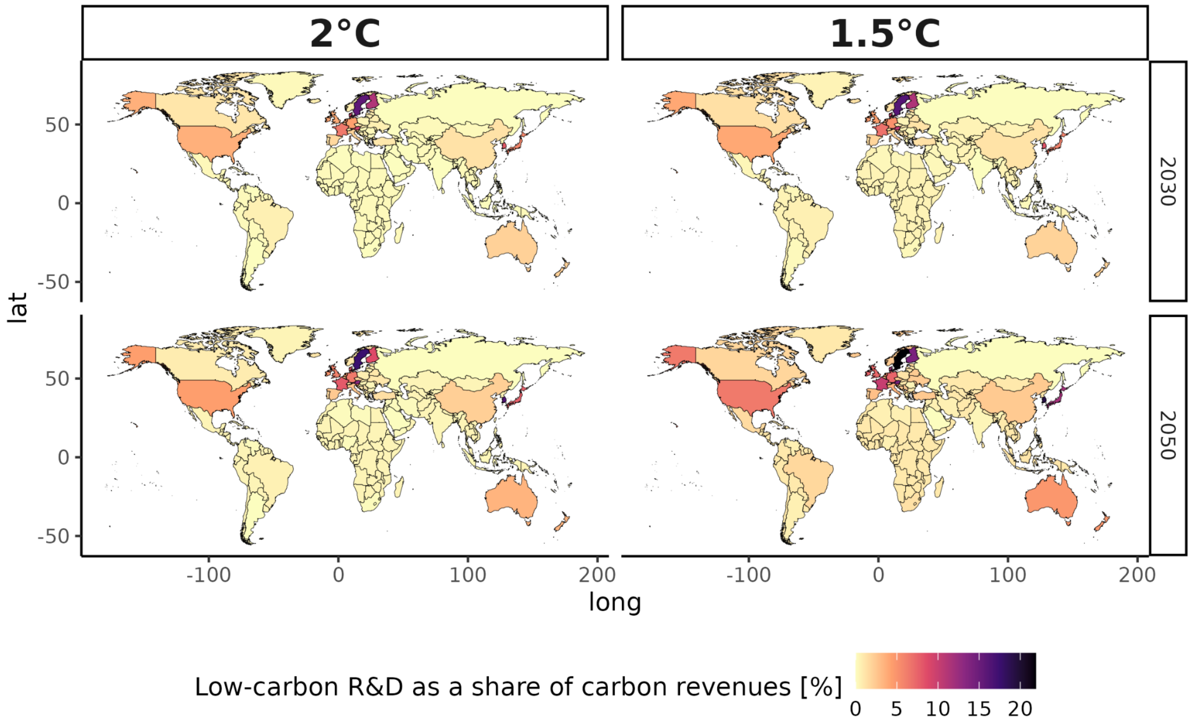

Supplementary figure 16 R&D Investment, including energy efficiency, as a share of carbon revenues in 2030 and 2050. Source: GEM-E3 model. Made with Natural Earth.

8.Global GHG pathways of all the scenarios .

Supplementary figure 17 shows the greenhouse gases (GHG) provided by the WITCH model. It shows the global GHG emissions for the REF and the two carbon policy scenarios. We observe an abrupt reduction of the emissions from 2020 onwards. Indeed, the forward looking planner anticipates very high carbon prices in the long run, and therefore chooses to decarbonize earlier. As illustrated by the difference between the full and the dashed line, if an optimal R&D strategy is implemented, decarbonisation efforts are anticipated. This effectively allows lower technology costs towards the end of the century. This is accompanied by an important scale-up of the R&D investment, particularly in CCS, energy efficiency, advanced biofuels and wind.

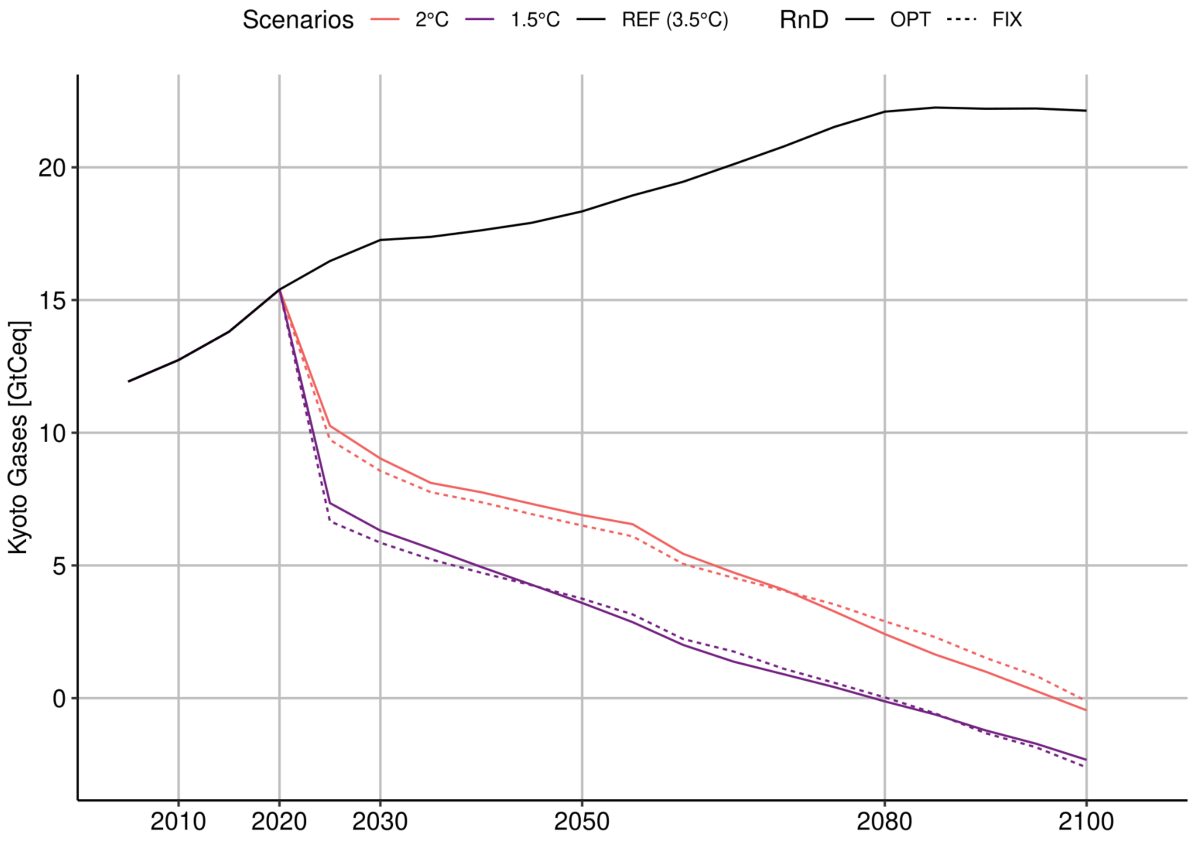

SUPPLEMENTARY FIGURE 17 GLOBAL PATHWAYS OF KYOTO GASES FOR THE DIFFERENT SCENARIOS CONSIDERED. SOURCE: WITCH MODEL.

9. Global energy demand

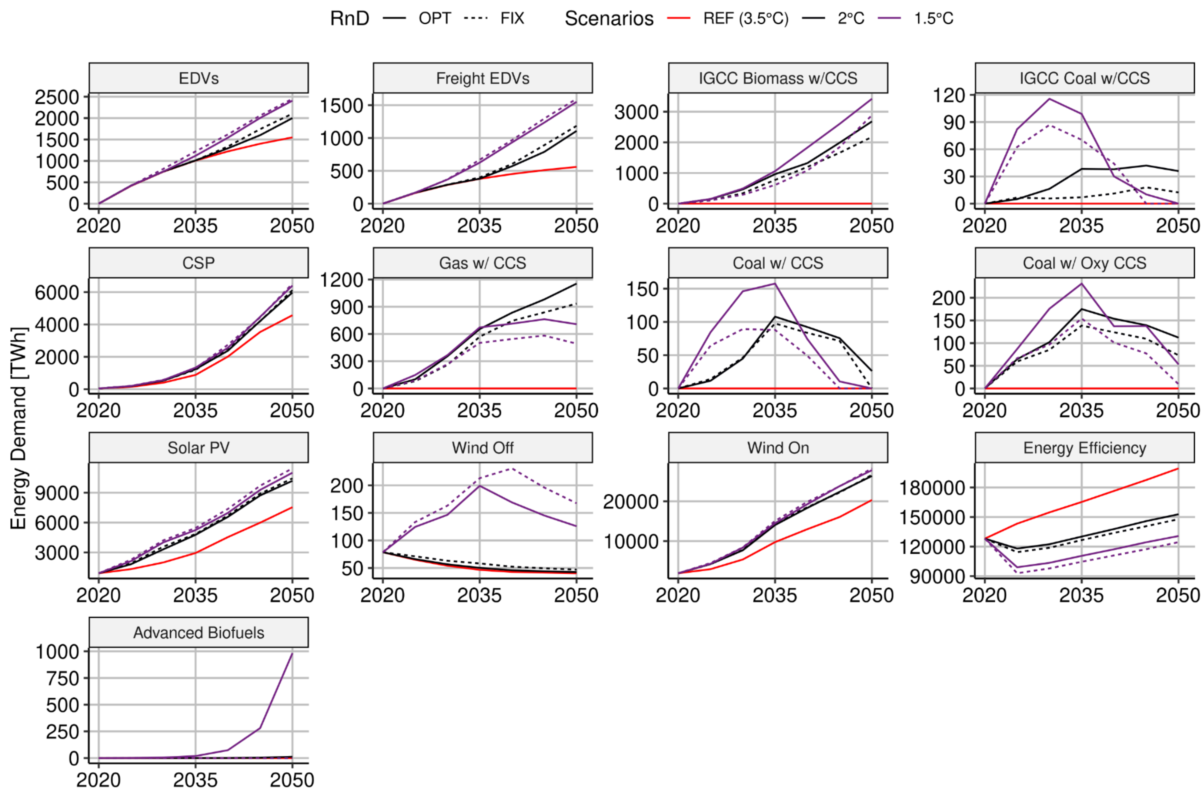

Supplementary figure 18 Global energy demand for all technologies form the WITCH model. IGCC stands for Integrated Gasification Combined Cycle and OPT for optimal R&D as opposed to FIX.

10. R&D Investment increase

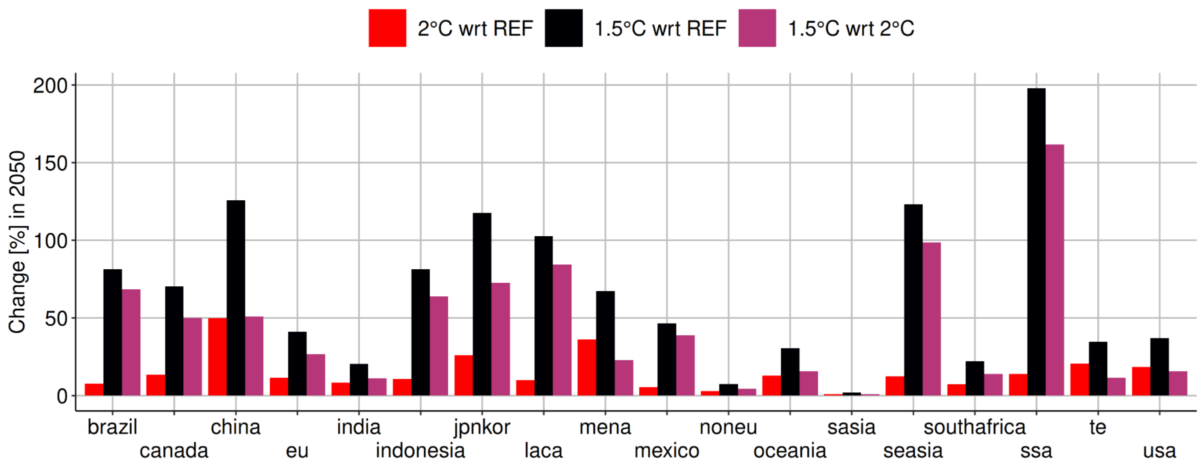

Supplementary figure 19 Cumulative R&D global investments, from 2020-2050, measured as changes relative to the reference (REF) and the 2°C scenarios. Source: WITCH model.

11. Comparison of Additional R&D investment fossil fuel subsidies GDP shares and current R&D investments

Supplementary figure 20 shows the comparison of current and estimated total R&D (including all sectors, i.e. not limited to low-carbon energy R&D), while supplementary figure 21 shows the current and estimated public low-carbon R&D investments.

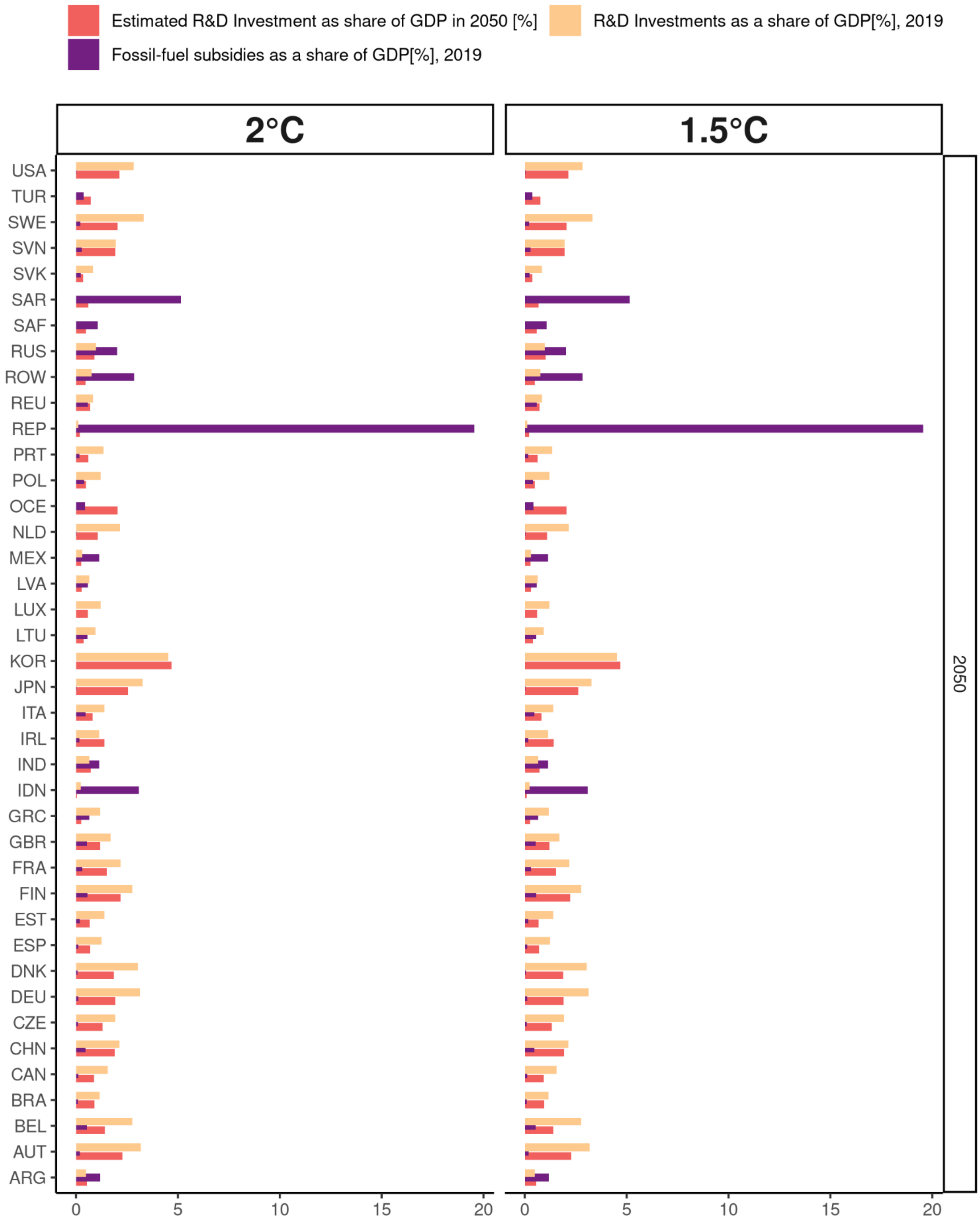

SUPPLEMENTARY FIGURE 20. GDP SHARE OF FOSSIL FUEL SUBSIDIES IN 2019 AND ESTIMATED ADDITIONAL R&D INVESTMENT ESTIMATED BY GEM-E3 PER SCENARIO INCLUDING ALL SECTORS. THE Y AXIS SHOWS THE GEM-E3 REGION CODES, DESCRIBED IN E3MODELLING (2017). IEA FOSSIL FUEL SUBSIDIES DATABASE, OECD.STAT- INVENTORY OF SUPPORT MEASURES FOR FOSSIL FUELS AND IMF ENERGY SUBSIDIES TEMPLATE (PRE-TAX CALCULATIONS) AND GDP, R&D INVESTMENT AND POPULATION FORM WDI.

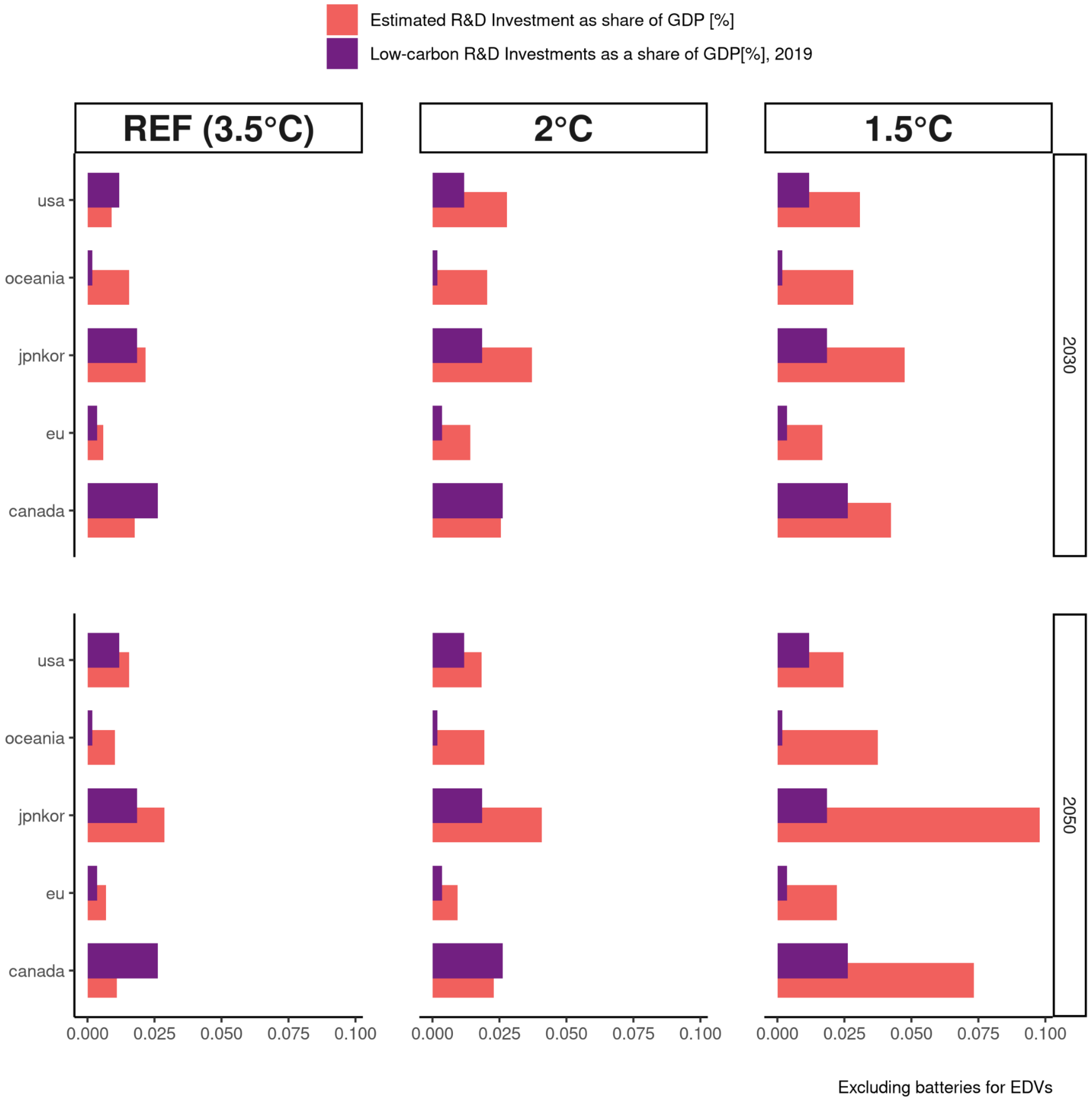

SUPPLEMENTARY FIGURE 21. LOW-CARBON PUBLIC R&D INVESTMENT ESTIMATED BY WITCH PER SCENARIO, EXCLUDING BATTERIES FOR EDVS. THE Y AXIS SHOWS THE WITCH REGION AS DESCRIBED IN SUPPLEMENTARY TABLE 3. 2019 DATA WAS TAKEN FROM IEA (2021).

12. R&D investment needs and climate finance.

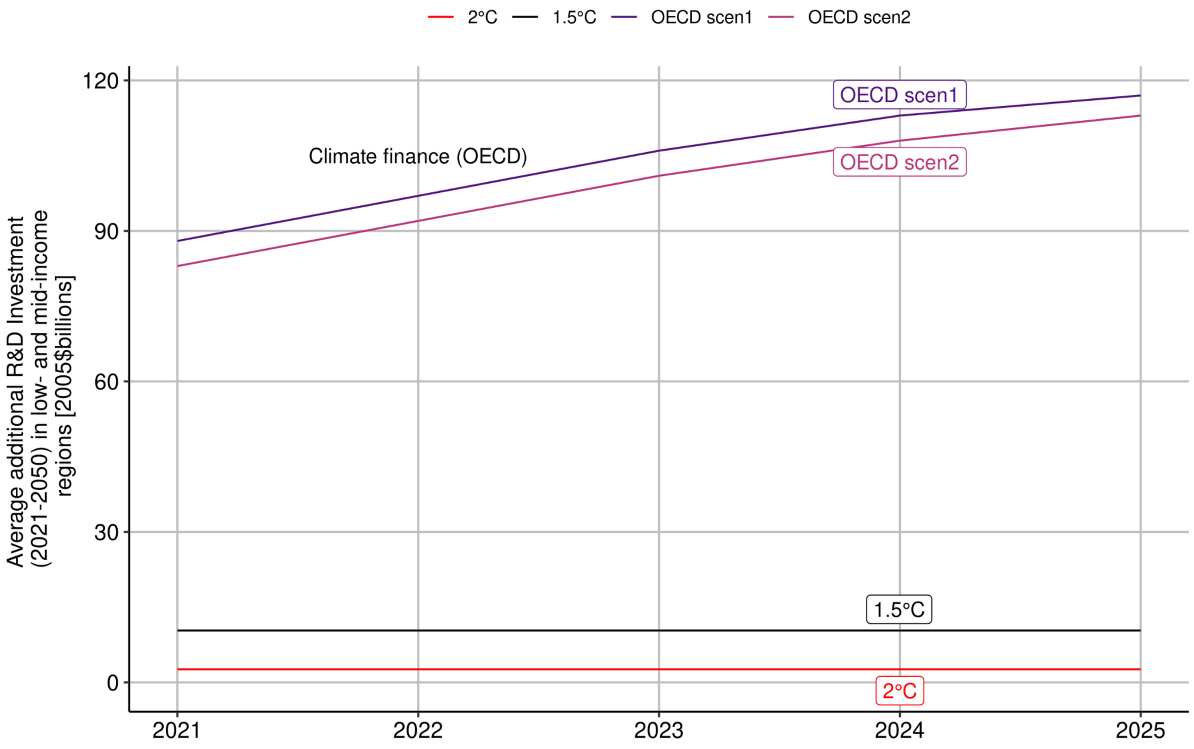

Supplementary figure 22. OECD 2021 climate finance scenarios compared with the estimated average additional R&D investment (2021-2050) needs in low- and mid-income countries for all the scenarios. In the low- and mid-income regions we have included brazil, china, india, indonesia, laca,mena ,mexico ,sasia , seasia, southafrica, ssa and te , as defined in Supplementary table 3.

Supplementary References

[1] T. Longden, “Travel intensity and climate policy: The influence of different mobility futures on the diffusion of battery integrated vehicles,” Energy Policy, vol. 72, p. 219–234, September 2014.

[2] V. Bosetti, C. Carraro, E. Massetti and M. Tavoni, “International energy R&D spillovers and the economics of greenhouse gas atmospheric stabilization,” Energy Economics, vol. 30, p. 2912–2929, November 2008.

[3] D. Popp, “ENTICE: endogenous technological change in the DICE model of global warming,” Journal of Environmental Economics and Management, vol. 48, p. 742–768, July 2004.

[4] G. Marangoni and M. Tavoni, “The clean energy R&D strategy for 2C,” Climate Change Economics, vol. 05, p. 1440003, February 2014.

[5] R. Kneller, “Frontier Technology, Absorptive Capacity and Distance,” Oxford Bulletin of Economics and Statistics, vol. 67, p. 1–23, February 2005.

[6] R. Griffith, S. Redding and J. V. Reenen, “R&D and Absorptive Capacity: Theory and Empirical Evidence,” Scandinavian Journal of Economics, vol. 105, p. 99–118, March 2003.

[7] I. Haščič and M. Migotto, "Measuring environmental innovation using patent data," 2015.

[8] G. Peri, "Determinants of Knowledge Flows and Their Effect on Innovation," The Review of Economics and Statistics, vol. 87, p. 308–322, 2005.

[9] E. Verdolini and M. Galeotti, "At home and abroad: An empirical analysis of innovation and diffusion in energy technologies," Journal of Environmental Economics and Management, vol. 61, p. 119–134, March 2011.

[10] IEA, RD&D Budget, Energy Technology RD&D Statistics (database), OECD Publishing, 2021.

[11] K. Mullen, D. Ardia, D. Gil, D. Windover and J. Cline, "DEoptim: An R Package for Global Optimization by Differential Evolution," Journal of Statistical Software, vol. 40, 2011.

[12] N. Kouvaritakis, A. Soria and S. Isoard, "Modelling energy technology dynamics: methodology for adaptive expectations models with learning by doing and learning by searching," International Journal of Global Energy Issues, vol. 14, pp. 104-115, 2000.

[13] L. Paroussos, K. Fragkiadakis and P. Fragkos, "Macro-economic analysis of green growth policies: the role of finance and technical progress in Italian green growth," Climatic Change, vol. 160, p. 591–608, September 2019.

[14] K. Fragkiadakis, L. Paroussos and P. Capros, "D4.3.2: Technical description of the R&I module of GEM-E3-RD model," https://www.monroeproject.eu/wp-content/uploads/2019/05/D4.3.2-Technical-description-of-the-RI-module-of-GEM-E3-RD-model.pdf, 2019.

[15] J. Ma, Z. Y. Stringer. D. and S. Kim, The Breakneck Rise of China's Colossus of Electric-Car Batteries, 2018.

[16] E. C. European Commission, "Building a globally competitive batteries manufacturing value chain in Europe.," https://ec.europa.eu/commission/sites/beta-political/files/factsheet-competitive-batteries-value-chain_april2019.pdf, 2019.

[17] A. Fiorini, F. Pasimeni, A. Georgakaki and E. Tzimas, "Analysis of the European CCS Research and Innovation Landscape," Energy Procedia, vol. 114, p. 7651–7658, July 2017.

[18] V. Krey, F. Guo, P. Kolp, W. Zhou, R. Schaeffer, A. Awasthy, C. Bertram, H.-S. de Boer, P. Fragkos, S. Fujimori, C. He, G. Iyer, K. Keramidas, A. C. Köberle, K. Oshiro, L. A. Reis, B. Shoai-Tehrani, S. Vishwanathan, P. Capros, L. Drouet, J. E. Edmonds, A. Garg, D. E. H. J. Gernaat, K. Jiang, M. Kannavou, A. Kitous, E. Kriegler, G. Luderer, R. Mathur, M. Muratori, F. Sano and D. P. van Vuuren, "Looking under the hood: A comparison of techno-economic assumptions across national and global integrated assessment models," Energy, vol. 172, p. 1254–1267, April 2019.

[19] J.-F. Mercure, F. Knobloch, H. Pollitt, L. Paroussos, S. S. Scrieciu and R. Lewney, "Modelling innovation and the macroeconomics of low-carbon transitions: theory, perspectives and practical use," Climate Policy, vol. 19, p. 1019–1037, June 2019.

[20] L. Clarke, J. Weyant and J. Edmonds, "On the sources of technological change: What do the models assume?," Energy Economics, vol. 30, p. 409–424, March 2008.

[21] B. Nagy, J. D. Farmer, Q. M. Bui and J. E. Trancik, “Statistical Basis for Predicting Technological Progress,” PLoS ONE, vol. 8, p. e52669, February 2013.

[22] E. Verdolini, L. D. Anadón, E. Baker, V. Bosetti and L. A. Reis, “Future Prospects for Energy Technologies: Insights from Expert Elicitations,” Review of Environmental Economics and Policy, vol. 12, p. 133–153, January 2018.

[23] G. Anandarajah and W. McDowall, “Multi-cluster Technology Learning in TIMES: A Transport Sector Case Study with TIAM-UCL,” in Lecture Notes in Energy, Springer International Publishing, 2015, p. 261–278.

[24] A. Elia, M. Kamidelivand, F. Rogan and B. Ó. Gallachóir, “Impacts of innovation on renewable energy technology cost reductions,” Renewable and Sustainable Energy Reviews, vol. 138, p. 110488, March 2021.

[25] K. Fragkiadakis, P. Fragkos and L. Paroussos, “Low-Carbon R&D Can Boost EU Growth and Competitiveness,” Energies, vol. 13, p. 5236, October 2020.

[26] E. S. Rubin, I. M. L. Azevedo, P. Jaramillo and S. Yeh, “A review of learning rates for electricity supply technologies,” Energy Policy, vol. 86, p. 198–218, November 2015.
